# Effects of whey protein on glycemic control and serum lipoproteins in patients with metabolic syndrome and related conditions: a systematic review and meta-analysis of randomized controlled clinical trials

**DOI:** 10.1186/s12944-020-01384-7

**Published:** 2020-09-21

**Authors:** Elaheh Amirani, Alireza Milajerdi, Željko Reiner, Hamed Mirzaei, Mohammad Ali Mansournia, Zatollah Asemi

**Affiliations:** 1 Research Center for Biochemistry and Nutrition in Metabolic Diseases, Institute for Basic Sciences, Kashan University of Medical Sciences, Kashan, Iran; 2 Students’ Scientific Research Center, Tehran University of Medical Sciences, Tehran, Iran; 3 Department of Community Nutrition, School of Nutritional Sciences and Dietetics, Tehran University of Medical Sciences, Tehran, Iran; 4 Department of Internal Medicine, University Hospital Centre Zagreb, School of Medicine, University of Zagreb, Zagreb, Croatia; 5 Department of Epidemiology and Biostatistics, School of Public Health, Tehran University of Medical Sciences, Tehran, Iran

**Keywords:** Whey protein, Insulin resistance, Metabolic syndrome, Triglycerides, Total cholesterol, LDL-cholesterol, HDL-cholesterol

## Abstract

**Background:**

This systematic review and meta-analysis aimed to assess the effects of whey protein on serum lipoproteins and glycemic status in patients with metabolic syndrome (MetS) and related disorders.

**Methods:**

Online databases, such as Web of Science, Cochrane Library, PubMed and Scopus were systematically searched by two independent authors from inception until 30th April 2020 for English randomized clinical trials investigating the efficacy of whey protein administration in subjects with Mets or related conditions on the parameters of glycemic and lipid control compared to certain control. In order to evaluate the included studies’ methodological quality, Cochrane Collaboration risk of bias tool was applied. Using Cochrane’s Q test and I-square (I^2^) statistic, the included trials’ heterogeneity was also examined. Using a random-effects model, data were pooled, and weighted mean difference (WMD) was considered as the overall effect size.

**Results:**

Twenty-two studies were selected to be included in this meta-analysis. Consumption of whey protein resulted in significant reduction of HbA1c (WMD: -0.15; 95% CI: − 0.29, − 0.01) insulin (WMD: -0.94; 95% CI: − 1.68, − 0.21) and homeostasis model assessment-estimated insulin resistance (HOMA-IR) (WMD: -0.20; 95% CI: − 0.36, − 0.05). A significant reduction in triglycerides levels (WMD: -17.12; 95% CI: − 26.52, − 7.72), total cholesterol (WMD: -10.88; 95% CI -18.60, − 3.17), LDL-cholesterol levels (WMD: -8.47 95% CI: − 16.59, − 0.36) and total cholesterol/HDL-cholesterol ratio (WMD: -0.26; 95% CI: − 0.41, − 0.10) was found as well.

**Conclusions:**

This meta-analysis suggests that supplementation with whey protein had beneficial effect on several indicators of glycemic control and lipid parameters in patients with MetS and related conditions.

## Background

Obesity, atherogenic dyslipidemia, arterial hypertension (HTN) and insulin resistance are the most important risk factors of cardiovascular disease (CVD). Often there is a clustering of these risk factors in one patient which is then called metabolic syndrome (MetS). MetS increases also the risk of type 2 diabetes mellitus (T2DM) [1]. It is estimated that over 20% of adults in Western countries have MetS with a clear tendency to increase [2, 3]. Many studies in healthy populations as well as in patients have reported that higher dairy consumption decreases the risk of MetS or some of the components of MetS and diabetes [4, 5]. It has been documented that specific components of dairy, including calcium, other minerals, and proteins such as whey proteins and casein [6], may have favorable effects on these risk factors.

There are different types of whey protein such as concentrate, isolate, hydrolysate and native whey protein, which come in multiple formulations including milk, milk powder and specialized formula with a higher content of certain amino acids [7]. This protein seems to have anti-inflammatory effects, beneficial effects on immunity, blood pressure and cholesterol as well as some anticancer properties [8]. Some favorable metabolic effects of whey protein may result from increasing the release of hormones including glucagon like-peptide 1 (GLP-1), leptin, and cholecystokinin, and the reduction of ghrelin and therefore the result might be weight reduction. Biological benefits of whey protein also might be associated to its nutritional components, especially cysteine and branched-chain amino acids (BCAAs). Whey protein also stimulates immune function, immunoglobulins and antioxidants [7].

The effects of whey protein on glycemic control and serum lipoproteins are controversial. In a study in which patients with MetS were taking yogurt fortified with whey protein during 10-weeks, it significantly reduced triglycerides levels and insulin resistance, and significantly increased HDL-cholesterol levels [9]. Supplementation with whey proteins during 12 weeks in overweight and obese subjects was associated with a significant decrease in total cholesterol and LDL- cholesterol and an improvement in fasting insulin concentrations and homeostasis model assessment of insulin resistance (HOMA-IR) scores [10]. In a meta-analysis by Wirunsawanya et al. [11], which included trials on overweight and obese patients, whey protein administration improved some CVD risk factors such as systolic and diastolic blood pressure, fasting plasma glucose (FPG), HDL-cholesterol, and total cholesterol levels, but did not influence other metabolic parameters.

The results of different studies which analyzed the impact of different types and amounts of whey protein on metabolic parameters were controversial. The aim of this systematic review and meta-analysis was to analyze the current information concerning whey protein effects on serum lipoproteins and glycemic control in patients with MetS and associated disorders like HTN, obesity, and diabetes mellitus.

## Materials and methods

### Search strategies and selected outcomes

Protocol of study was registered in international prospective register of systematic reviews (PROSPERO) (ID: CRD42020203067). In order to find and include relevant investigations published from inception until 30th April 2020, international databases, such as Web of Science, PubMed, Scopus and Cochrane Library were searched for studies evaluating the effects of whey protein supplementation among patients with MetS and associated disorders. PROSPERO database was searched to identify similar records. The strategy of search and keywords are presented in Supplemental file- Table 1; This meta-analysis was conducted to determine the efficacy of whey protein on the following outcomes: parameters of glycemic control including fasting plasma glucose (FPG), fasting insulin levels, HOMA-IR, Hemoglobin A1c (HbA1c), and lipid profiles including triglycerides levels, total-, high density lipoprotein (HDL-), low density lipoprotein (LDL-), and very density lipoprotein (VLDL-) cholesterol levels in fasting state and the total/HDL-cholesterol ratio.

### Inclusion and exclusion criteria

In this meta-analysis, randomized controlled trials (RCTs) which fulfilled the following criteria for participants, interventions, comparisons, outcomes, and study design (PICOS) were included: 1) Participants: human subjects with MetS or conditions related to this syndrome. 2) Intervention: whey protein administration. 3) Comparisons: control, including placebo, carbohydrate supplementation, usual diet or no intervention. 4) Outcomes: serum lipoproteins and glycemic status. 5) Study design: parallel or cross-over design. In addition, data need to be presented as mean/median with standard deviation (SD) or standard error (SE) or related 95% confidence intervals (CIs) or interquartile range (IQR) for both intervention and control groups. Relevant articles which were written English were included. Inclusion criteria for MetS were: 3 or more of these parameters - increased waist circumference (according to specific cut point for population), triglycerides levels ≥150 mg/dl, blood pressure ≥ 130/85 mmHg, FPG concentrations ≥100 mg/dl, and HDL-cholesterol values < 40 mg/dl for men and < 50 mg/dl for women [12]. Dyslipidemia, overweight and obesity (BMI ≥ 25), insulin resistance, diabetes, HTN, polycystic ovary syndrome (PCOS), non-alcoholic fatty liver disease, and CVD were considered as conditions related to MetS. Studies that compared whey protein with other protein supplements (casein, gelatin and etc.), trials without control group, case reports, observational studies, animal experiments and in vitro studies were excluded. Concerning studies designed to analyze exercise training, those which compared whey protein effects against exercise also were excluded.

### Data extraction and quality assessment

Based on the eligibility criteria, two authors (HM and EA) independently screened the articles. At the beginning, studies’ abstracts and titles were reviewed. As the second step, to ensure that a study is suitable for this meta-analysis, relevant articles’ full-text was evaluated. In case of relevant studies with incomplete data or without full text, a request was emailed to correspond author. Any disagreement was resolved by the judgment of the third author (ZA).

The following data were extracted from selected trials: the authors’ name, study duration, whey protein type and dosage, exercise training, study design, study location, sample size, publication year, the type of the disease, the SD and mean for serum lipoproteins and glycemic control in each treatment group. For incorporating cross-over trials which reporting data of each period separately, only data from the first period were included [13]. For studies presenting median and IQR, mean was estimated by (first quartile + third quartile)/2, and SD was estimated by (third quartile – first quartile)/1.35 [14]. For studies presenting 95% CI, SE was estimated by (upper limit – lower limit)/3.92 and SD was calculated as SE × √n [15]. Unit conversion of mmol/L to mg/L was done using Units lab online data base [16]. Concerning a previous meta-analysis by Guasch-Ferré et al. [17], three categories based on control group of the included studies were considered: 1) placebo product, 2) non-intervention control like usual diet or no supplementation, and 3) carbohydrate supplementation like maltodextrin or sugar. These categories were used to explore the potential heterogeneity due to different types of controls. Quality of Included RCTs was evaluated by same independent authors using Cochrane tool. In addition, the quality of findings was assessed using Grading of Recommendations Assessment, Development and Evaluation (GRADE) approach.

### Data synthesis and statistical analysis

Whey protein effects on the alterations of the analyzed parameters were calculated. For pooling data to determine effect sizes, weighted mean difference (WMD) with 95% CI was utilized. The change score method was used to calculate the effect size of whey protein on the analyzed parameters. The fixed-effect model was used to report the pooled effect sizes using 95% CI. In cases of high between-study heterogeneity, we used random-effect model to analyze data. Furthermore, meta-regression was done to explore any dose-response association between outcomes of interest and duration of supplementation.

### Heterogeneity and publication bias

Heterogeneity of included studies was evaluated using Cochrane’s Q test and I-square test (I^2^ greater than 50% showing significant heterogeneity) [18, 19]. In cases of high between-study heterogeneity, we stratified the included studies based on participants’ age to studies that recruited subjects with a mean age of 20–65 years (exclusively adults) and those done on subjects aged ≥20 (including both adults and elderly subjects). In addition, a subgroup analysis was done concerning the participants’ health condition taking into the consideration studies on healthy participants and studies on patients with any chronic disease, including diabetes, CVD, and cancers. The other subgroup analyses were done based on intervention type (whey protein/isolated whey protein), study duration (< 12 weeks/≥12 weeks), and study sample size (*n* < 50/*n* ≥ 50). The cut-points for the study duration and sample size were selected based on sufficient number of studies which were included in each subgroup. In order to assess the effects of heterogeneity on outcomes, 95% predictive intervals (PI) also were estimated manually [20]. Publication bias was evaluated by the funnel plot and tested for statistical significance using the Egger’s test [21]. Both STATA 11.0 (Stata Corp., College Station, TX) and Review Manager 5.3 (Cochrane Collaboration, Oxford, UK) were applied for data analysis.

## Results

### Characteristics of included studies

**Fig. 1:**
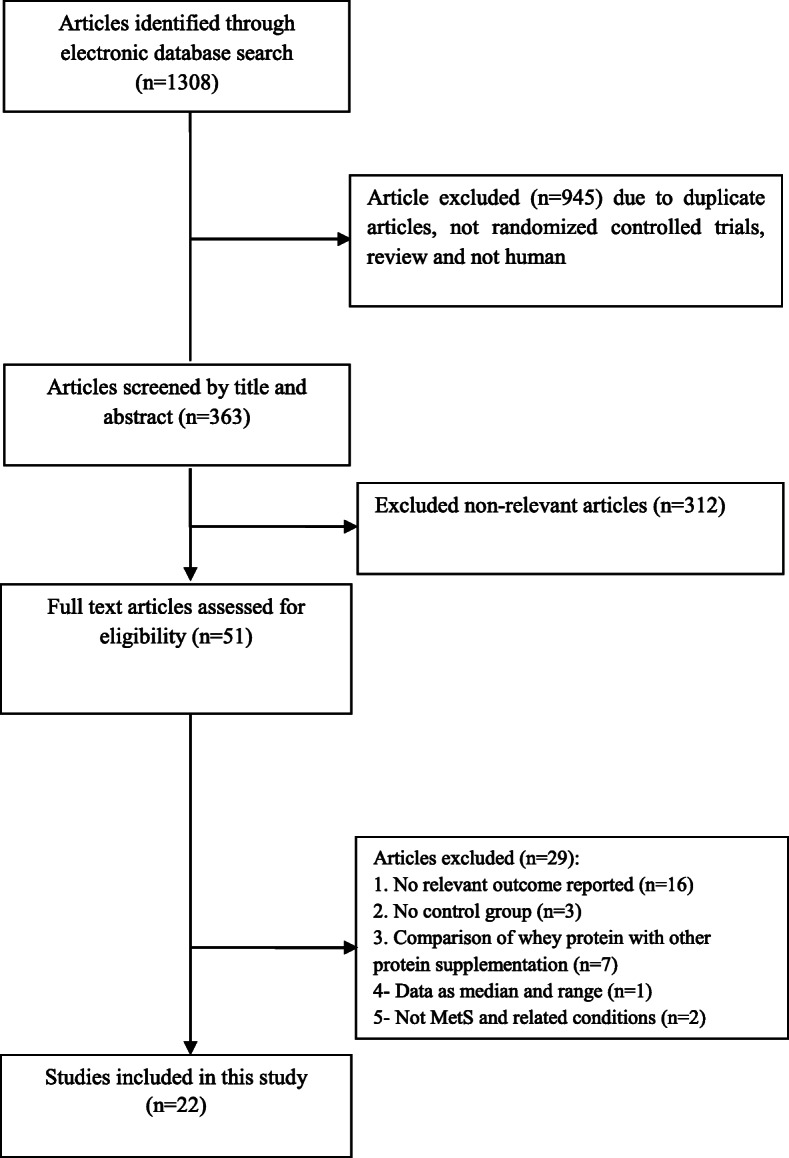
Literature search and review flowchart for selection of studies

Twenty-two studies were included in this systematic review and meta-analysis. These studies were published between 2007 and 2019. Flow-diagram for study selection is shown in Fig. 1**.** One thousand one hundred three subjects, 576 in intervention and 527 in control groups, were enrolled in included studies. Characteristics of included studies are summarized in Table 1. Participants had chronic diseases like HTN [22, 28, 31, 40] and T2DM [33, 36, 38] in some studies. The intervention period varied from 4 weeks to 24 weeks. Whey protein supplements were administrated in dosages which varied from 70 mg/d to 90 g/d. In 5 studies, a dosage of ≤20 mg/d was used [9, 23, 28, 29, 32]. Six trials used whey protein in dosages between 20 and ≤ 40 mg/d [25, 30, 33, 36, 38, 40]. Five other studies used it at a daily dose between 40 and 60 mg [10, 26, 31, 35, 41]. Moreover, a dosage ≥60 mg/d of whey protein was used in one study [27]. Whey protein also was provided at daily dosages of 0.4 g/kg [39] and 0.5 g/kg body weight [34] in other studies. Three studies did not report the amount of whey protein used in their interventions [22, 24, 37]. Eight studies used isolate [10, 24, 27, 28, 31, 37–39] and 4 studies used concentrate whey protein [33, 34, 36, 40]. Combined isolate plus concentrate [30], hydrolysate [41], and intact [23] whey protein was reported each in one trial. Other studies did not report the type of whey protein.

**Table 1:** Characteristics of included studies

Authors	Year	Sample size (intervention vs. control)	Country, population and BMI (intervention vs. control)	Gender and M/F number	Exercise	Intervention (name and daily dose)	Type/formulation of WP	Control (type, name and daily dose)	Duration (week)	Age range (y)	Present data	Results
Lee et al. [22]	2007	27/26	Germany/ Subjects with mild hypertension BMI: 28.5 ± 4.2, 27.2 ± 4.0	Both 14/13, 16/10	No	Whey peptides supplemented milk drink	NR	Placebo product: Non-supplemented milk drink	12	30–65	FPG Insulin HOMA-IR TG TC HDL-C LDL-C	No significant change in FPG, insulin, HOMA-IR, TG, TC, HDL-C and LDL-C between groups.
Frestedt et al. [23]	2008	31/28	USA/ Obese subjects on energy reduction BMI: 35.7 ± 0.7, 35.4 ± 0.7	Both NR	No	20 g/d WP and peptides from a specialized supplement (Prolibra™)	Intact + peptides	CHO supplementation: beverage containing maltodextrin	12	25–50	TG TC HDL-C LDL-C	TC decreased in intervention group, but no significant change in TG, TC, HDL-C and LDL-C between groups
Kasim-Karakas et al. [24]	2009	11/13	California/ Overweight or obese women with PCOs on energy reduction BMI: 38.9 ± 2.1, 35.4 ± 1.2	F	No	Sugar-free WP providing 240 kcal	Isolate	CHO supplementation: glucose plus maltose and providing 240 kcal	8	18–45	FPG, Insulin HOMA-IR HbA1c TG TC HDL-C	TC and HDL-C decreased significantly. No significant change in FPG, insulin, HOMA-IR and TG between groups.
Denysschen et al. [25]	2009	9/9	USA/ Overweight men BMI: 28.5 ± 2.3, 27.9 ± 1.44	M	Yes	26.6 g/d WP	NR	CHO supplementation: 25 g/d complex carbohydrate	12	21–50	TG TC HDL-C TC/HDL-C ratio	TC decreased in both groups, but no significant change in TG, TC, HDL-C and TC/HDL-C ratio between groups
Claessens et al. [26]	2009	18/16	Netherlands/ Overweight or obese subjects BMI: 33.4 ± 4.2, 32.4 ± 4.8	Both 6/12, 6/10	No	50 g/d WP	NR	CHO supplementation: 50 g/d maltodextrin	12	30–60	FPG Insulin HOMA-IR HbA1c TG TC HDL-C LDL-C	TC, HDL-C and LDL-C decreased in both groups.
Pal et al. [10]	2010	25/25	Australia/ Overweight or obese subjects BMI: 32.0 ± 4.0, 30.6 ± 4.5	Both NR	No	54 g/d WP	Isolate	CHO supplementation: 54 g/d glucose	12	18–65	FPG Insulin HOMA-IR TG TC HDL-C LDL-C	Insulin, HOMA-IR, TG, TC and LDL-C decreased significantly, but no significant change in FPG.
Sheikholeslami Vatani and Ahmadi Kani Golzar [27]	2012	9/10	Iran/ Overweight young men BMI: 26.5 ± 1.2, 27.2 ± 1.6	M	Yes	90 g/d WP	Isolate	Placebo product: 90 g/d placebo	6	23 ± 2, 21 ± 1	FPG TG TC HDL-C LDL-C	LDL-C and TG decreased in both groups and TC decreased in intervention group and HDL-C increased in intervention group, but No significant change between groups.
Petyaev et al. [28] (a)	2012	10/5	Russia/ Subject with prehypertension BMI: 25.9 *±* 2.8, 26.8 *±* 5.7	Both 6/4, 3/2	No	70 mg/d WP	Isolate	Placebo product: Placebo pills	4	45–73	TG TC HDL-C LDL-C	No significant changes in both groups.
Petyaev et al. [28] (b)	2012	10/5	Russia/ Subject with prehypertension BMI: 27.2 *±* 3.4, 26.8 *±* 5.7	Both 5/5, 2/3	No	70 mg/d WP + 7 mg/d lycopene	Isolate	Placebo product: Placebo pills	4	45–73	TG TC HDL-C LDL-C	TG, TC and LDL-C significantly reduced and HDL-C significantly increased in intervention group.
Tovar et al. [29]	2012	44/44 sex	Sweden/ Overweight and obese subjects BMI: 28.5 ± 2	Both 8/36	No	4.3 g/d WP powder as an ingredient in a multifunctional diet	NR	Non-intervention control: control diet	4	50–73	FPG Insulin HOMA-IR HbA1c TG TC HDL-C LDL-C	FPG significantly increased and insulin, HbA1c, TG, TC, LDL-C and HDL-C significantly decreased in intervention group. Between group changes were significant for HbA1c, TG, TC and LDL-C
Ormsbee et al. [30]	2015	13/10	USA/Sedentary overweight/obese women BMI: 34.4 *±* 4.7, 33.1 *±* 5.4	F	Yes 3 days weekly	30 g/d WP powder	Isolate + concentrate	CHO supplementation: 34 g/d maltodextrin powder	4	18–45	FPG Insulin HOMA-IR TG TC HDL-C LDL-C	No significant changes in both groups.
Fekete et al. [31]	2016	38/38 Both sex	United Kingdom/ Subjects with prehypertension and mild HTN BMI: 27.1 ± 4.93	Both 20/18, 20/18	No	56 g/d WP	Isolate	CHO supplementation: 54 g/d maltodextrin	8	30–77	FPG Insulin HOMA-IR TG TC HDL-C LDL-C TC/HDL-C ratio	TG and TC significantly decreased compared control group.
Tovar et al. [32]	2016	23/24	Sweden/ Overweight and obese subjects BMI: 28.00 ± 0.09, 27.7 ± 2.44	Both 3/20, 9/15	No	4.3 g/d WP powder as an ingredient in a multifunctional diet	NR	Non-intervention control: control diet	8	51–72	FPG Insulin HOMA-IR HbA1c TG TC HDL-C LDL-C	TC and LDL-C significantly decreased compared control group.
Jakubowicz et al. [33]	2017	17/15	Venezuela/ T2DM BMI: 32.2 ± 0.87, 32.1 ± 1.27	Both NR	No	Breakfast containing 28 g/d WP	80% concentrate	CHO supplementation: high-carbohydrate breakfast containing 17 g protein from various sources	12	59 ± 4.84	FPG HbA1c	FPG and HbA1c significantly decreased in both groups and between group changes were significant compared control.
Lopes Gomes et al. [34]	2017	15/15	Brazil/ Women who regained at after a Roux-en-Y gastric bypass on energy reduction BMI: 36 ± 6, 35 ± 4	F	No	WP at a dosage of 0.5 g/kg of ideal body weight	Concentrate	Non-intervention control: hypocaloric diet with normal protein	16	≥18	FPG HOMA-IR TG TC HDL-C LDL-C	TC, LDL-C and HDL-C significantly decreased in both groups
Kjølbæk et al. [35] (a)	2017	39/19	Denmark/ Overweight and obese subjects on weight maintenance period after a weight loss period BMI: 33.2 ± 3.31	Both NR	No	45 g/d WP powder	High a-lactalbumin	CHO supplementation: 48 g/d maltodextrin powder	24	18–60	FPG Insulin HOMA-IR TG TC HDL-C LDL-C	No significant changes compared control.
Kjølbæk et al. [35] (b)	2017	38/19	Denmark/ Overweight and obese subjects on weight maintenance period after a weight loss period BMI: 33.2 ± 3.31	Both NR	No	45 g/d WP powder	High a-lactalbumin	CHO supplementation: 48 g/d maltodextrin powder	24	18–60	FPG Insulin HOMA-IR TG TC HDL-C LDL-C	No significant changes compared control.
Watson et al. [36]	2018	37/42	New Zealand/ T2DM BMI: 30.3 ± 5.5, 29.7 ± 4.5	Both 23/14, 21/21	No	Shake containing 34 g/d WP + 10 g/d guar	Concentrate	Placebo product: shake with 20 ml/d of a liquid raspberry	12	18–75	HbA1c	HbA1c significantly decreased compared control.
Kemmler et al. [37]	2018	33/34	Germany/ Older men with sarcopenic obesity BMI: 26.3 ± 2.5, 26.0 ± 2.5	M	No	WP supplement in order to realize a total daily protein amount of 1.7–1.8 g/kg body mass	Isolate	Non-intervention control	16	≥70	TG TC/HDL-C ratio	TC/HDL-C ratio significantly decreased in intervention group and was differed from control. No significant changes in TG.
Gaffney et al. [38]	2018	12/12	New Zealand/ T2DM men BMI: 29.6 ± 2.7, 30.1 ± 4.9	M	Yes 4–5 days weekly	Beverage containing WP 40 g/each exercise session	Isolate	CHO supplementation: beverage containing carbohydrate 60 g/each exercise session	10	53.5 ± 5.6, 57.8 ± 5.2	FPG HOMA-IR	FPG and HOMA-IR decreased in intervention group, but changes were not significant compared control.
Larsen et al. [39]	2018	14/15	Denmark/ Overweight and obese subjects on energy reduction BMI: 34.9 ± 5.12, 35.1 ± 5.71	Both NR	Yes 5 days weekly	0.4 g/kg WP supplement	Isolate	Non-intervention control: no supplementation	4	21–55	FPG Insulin TC	FPG significantly decreased in control group. Insulin and TC significantly decreased in both group with no significant between group changes.
Mohammadi-Sartang et al. [9]	2018	44/43	Iran/ Overweight/obese subjects with metabolic syndrome (BMI: 25–34.9) on energy reduction BMI: 30.1 ± 2.6, 30.8 ± 2.2	Both 17/27, 17/26	No	Fortified yogurt containing 10 g/d WP, 1000 mg calcium, and 1000 IU vitamin D	NR	Placebo product: low-fat conventional yogurt	10	20–65	FPG Insulin HOMA-IR TG TC HDL-C LDL-C	HOMA-IR and TG significantly decreased and HDL-C significantly increased in both groups and between group changes were significant compared control.
Yang et al. [40] (a)	2019	12/12	China/ Overweight subjects with prehypertension and mild HTN BMI: NR	Both NR	No	30 g/d WP powder	concentrate	CHO supplementation: 30 g/d maltodextrin powder	12	≥18	FPG TG TC HDL-C LDL-C	No significant changes compared control.
Yang et al. [40] (b)	2019	15/15	China/ Normal weight subjects with prehypertension and mild HTN BMI: NR	Both NR	No	30 g/d WP powder	concentrate	CHO supplementation: 30 g/d maltodextrin powder	12	≥18	FPG TG TC HDL-C LDL-C	No significant change compared control.
Rakvaag et al. [41] (a)	2019	15/16	Denmark/Subjects with abdominal obesity BMI: 28.4 ± 4.1, 30.3 ± 4.5	Both 9/6, 8/8	No	60 g/d whey protein + low fiber product	Hydrolysate	CHO supplementation: 60 g/d maltodextrin + low fiber product	12	≥40	FPG Insulin TG TC HDL-C LDL-C	TG and TC significantly decreased in intervention group. HDL-C significantly increased in intervention group.
Rakvaag et al. [41] (b)	2019	17/17	Denmark/Subjects with abdominal obesity BMI: 29.6 ± 2.3, 29.1 ± 3.6	Both 7/10, 7/10	No	60 g/d whey protein + high fiber product	Hydrolysate	CHO supplementation: 60 g/d maltodextrin + high fiber product	12	≥40	FPG Insulin TG TC HDL-C LDL-C	FPG significantly increased in intervention group. TC, LDL-C and HDL-C significantly increased in control group.

HOMA IR, homeostasis model assessment of insulin resistance; HbA1c, glycated hemoglobin; TG, triglycerides; TC, total cholesterol, HDL-C, HDL-cholesterol; LDL-C, LDL-cholesterol. WP, whey protein. CHO, carbohydrate. F, female. M, male. HTN, hypertension

Among studies analyzed in this meta-analysis concerning the significance of between group changes for glycemic parameters, significant decrease of FPG was reported in one study [33], while it was unaffected by treatment in 12 studies [9, 22, 24, 27, 30–32, 34, 35, 38, 40, 41], and it was increased in two studies [29, 39]. A significant decrease of insulin was shown in one study [9], while it was unaffected by treatment in 9 studies [22, 24, 29–32, 34, 35, 38]. In addition, a significant decrease of HOMA-IR was demonstrated in one study [9], while it was unaffected by treatment in 9 studies [22, 24, 29–32, 34, 35, 38]. A significant decrease of HbA1c was shown in 3 studies [29, 33, 36], while it was unaffected by treatment in 2 studies [24, 32]. However, 2 studies did not report the significance of between group changes for indicators of glycemic control [10, 26].

Among studies analyzed in this meta-analysis concerning the significance of between group changes for lipids and lipoproteins, a significant decrease of triglycerides was proven in 3 studies [9, 29, 31], and 2 effect sizes [41] (a) and [28] (b), while it was unaffected by treatment in 10 studies [22–25, 27, 30, 32, 34, 35, 37, 40], and 2 effect sizes [41] (b), and [28] (a). A significant decrease of total cholesterol was shown in 5 studies [24, 25, 29, 31, 32], and one effect size [28] (b), while it was unaffected by treatment in 10 studies [9, 22, 23, 27, 30, 34, 35, 39–41], and one effect size [28] (a). A significant decrease of LDL-cholesterol occurred in 2 studies [29, 32], and one effect size [28] (b), while it was unaffected by treatment in 11 studies [9, 22, 23, 25, 27, 30, 31, 34, 35, 40, 41], and one effect size [28] (a). A significant increase of HDL-cholesterol was shown in one study [9], and one effect size [28] (b), while it was unaffected by treatment in 12 studies [22–24, 27, 29–32, 34, 35, 40, 41], and decreased in one study [24]. A significant decrease of total−/HDL-cholesterol ratio was demonstrated in one study [37], while it was unaffected by treatment in two studies [25, 32]. However, 2 studies did not report the significance of between group changes for lipids and lipoproteins [10, 26].

### Quality assessment

In the present meta-analysis, the quality of included studies was assessed using Cochrane tool. Based on the components of quality assessment tool, 17 studies were at low risk in term of random sequence generation. For allocation concealment, 14 studies were found to be at low risk, also 13 studies were considered at low risk in term of blinding of participants and personnel. Five studies were at low risk in the aspect of blinding of outcome assessment. In addition, in term of incomplete outcome data, selective reporting and other sources of bias, 22, 15 and 17 studies were considered at low risk, respectively (Supplemental file- Table 2).

### The effects of whey protein on glycemic control

**Fig. 2:**
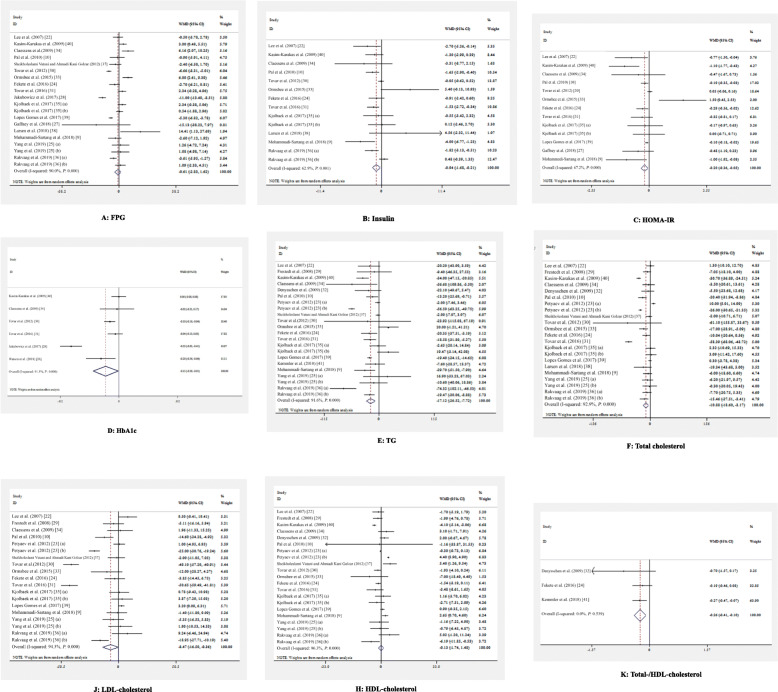
**a-k**. Meta-analysis of glycemic control and serum lipids. Weighted mean difference estimates for **a** FPG, **b** insulin, **c** HOMA-IR, **d** HbA1c, **e** triglycerides, **f** total cholesterol, **j** LDL-cholesterol, **h** HDL-cholesterol, and **k** total−/HDL-cholesterol in the whey protein and placebo groups (CI = 95%)

Consumption of whey protein resulted in significant reduction of insulin (12 studies with 14 effect sizes) (WMD: -0.94; 95% CI: − 1.68, − 0.21) (Table 2 & Fig. 2b), HOMA-IR (12 studies with 13 effect sizes) (WMD: -0.20; 95% CI: − 0.36, − 0.05) (Table 2 & Fig. 2c) and HbA1c (6 studies) (WMD: -0.15; 95% CI: − 0.29, − 0.01) **(**Table 2 & Fig. 2d). Whey protein intake did not have any effect on FPG (17 studies with 20 effect sizes) (WMD: -0.61; 95% CI: − 2.83, 1.62) (Table 2 & Fig. 2a). The quality of evidence was moderate for insulin and HbA1c in GRADE system. Also, FPG, HOMA-IR had a low evidence quality of evidence (Supplemental file- Table 3). After adjustment, PI indicated that results were insignificant for FPG (95% PI: − 3.05, 1.79), insulin (95%PI: − 1.91, 0.87), HOMA-IR (95%PI: − 0.60, 0.09) and HbA1c (95%PI: − 0.30, 0.01).

**Table 2:** The effects of whey protein intake on glycemic control and serum lipoproteins

Variables	Number of effect sizes	Weighted mean difference	CI 95%	Heterogeneity	
				I^2^ (%)	*P*- value heterogeneity
FPG	20	-0.61	−2.83, 1.62	90.0	< 0.001
HbA1C	6	−0.15	−0.29, − 0.01	91.3	< 0.001
Insulin	14	−0.94	−1.68, − 0.21	62.9	< 0.001
HOMA-IR	13	−0.20	−0.36, − 0.05	67.2	< 0.001
TC	22	−10.88	−18.60, −3.17	92.5	< 0.001
TG	22	−17.12	−26.52, −7.72	91.6	< 0.001
LDL	19	−8.47	−16.59, −0.36	94.3	< 0.001
HDL	21	−0.13	−1.74, 1.48	94.3	< 0.001
TC/HDL	3	−0.26	−0.41, − 0.10	00.0	0.53

*HOMA IR* homeostasis model assessment of insulin resistance, *HbA1c* glycated hemoglobin, *TG* triglycerides, *TC* total cholesterol, *HDL-C* HDL-cholesterol, *LDL-C* LDL-cholesterol

In the subgroup analysis of FPG, a significant change was seen in all subgroups except for participants’ age (adults) (WMD: -0.30; 95% CI: − 1.29, 0.69) and in studies which used isolated whey protein (WMD: 0.35; 95% CI: − 1.57, 2.28), placebo (WMD: -1.22; 95% CI: − 3.33, 1.43) and carbohydrate supplementation as control (WMD: -0.73; 95% CI: − 1.61, 0.15) **(**Table 3**)**. Whey protein also reduced HbA1c concentrations in all subgroups. Whey protein intake did not have any effect on insulin levels in studies performed on adults + elderly (WMD: -0.34; 95% CI: − 0.74, 0.07) and those which used a non-intervention controls (WMD: -0.30; 95% CI: − 0.81, 0.21). In a subgroup analysis of HOMA-IR, a significant change was seen in all subgroups except for studies with a duration < 12 weeks (WMD: -0.04; 95% CI: − 0.14, 0.06) and sample size ≥50 (WMD: -0.07; 95% CI: − 0.16, 0.01) or studies which used non-intervention controls (WMD: -0.06; 95% CI: − 0.12, 0.01).

**Table 3:** Subgroup analyses for the effects of whey protein intake on glycemic control and serum lipoproteins

Variables		Subgroups	Number of effect sizes	Pooled WMD	95% CI	I^2^ (%)	Between-study I^2^ (%)
FPG	Participants’ age	Adult	12	− 0.30	−1.29, 0.69	90.3	< 0.001
		Adult+Elderly	8	−3.79	−4.65, −2.93	86.0	
	Participants’ health condition	Healthy	12	−2.12	− 2.87, −1.37	90.9	0.40
		Chronic disease	8	−2.76	−4.05, −1.47	89.8	
	Intervention type	Isolated	6	0.35	−1.57, 2.28	76.8	< 0.01
		Whey proteins	14	−2.72	−3.43, − 2.01	92.0	
	Study duration	< 12 week	9	−1.62	−2.64, −0.60	90.6	0.09
		≥12 week	11	−2.74	−3.58, −1.89	90.3	
	Sample size	n < 50	13	−2.05	−2.89, −1.22	91.7	0.38
		n ≥ 50	7	−2.64	−3.67, −1.61	86.5	
	Type of control	Placebo product	3	−1.22	−3.33, 1.43	00.0	< 0.001
		Carbohydrate supplementation	13	−0.73	−1.61, 0.15	88.9	
		Non-intervention	4	−4.54	−5.56, −3.51	92.8	
HbA1C	Participants’ age	Adult	3	−0.15	−0.21, − 0.08	96.2	0.16
		Adult+Elderly	3	−0.09	−0.14, − 0.04	36.6	
	Participants’ health condition	Healthy	3	−0.08	−0.12, − 0.03	00.0	0.02
		Chronic disease	3	−0.17	−0.23, − 0.10	96.1	
	Study duration	< 12 week	3	−0.06	−0.10, − 0.01	49.4	< 0.001
		≥12 week	3	−0.33	−0.42, − 0.24	91.2	
	Sample size	n < 50	3	−0.11	−0.16, − 0.06	94.7	0.91
		n ≥ 50	3	−0.11	−0.17, − 0.05	20.1	
Insulin	Participants’ age	Adult	9	−1.43	−2.21, 0.65	48.0	0.01
		Adult+Elderly	5	−0.34	−0.74, 0.07	7.09	
	Participants’ health condition	Healthy	10	−0.39	−0.078, − 0.00	63.6	0.01
		Chronic disease	4	−1.67	−2.63, −0.70	33.4	
	Intervention type	Isolated	4	−1.15	−2.10, −0.20	67.2	0.09
		Whey proteins	10	−0.42	− 0.81, − 0.03	62.9	
	Study duration	< 12 week	7	−0.49	−0.95, − 0.03	68.5	0.59
		≥12 week	7	−0.69	−1.27, − 0.11	61.7	
	Sample size	n < 50	7	−0.60	−1.17, − 0.02	69.8	0.89
		n ≥ 50	7	−0.55	−1.01, − 0.09	60.3	
	Type of control	Placebo product	2	−3.30	−5.18, 1.42	00.0	0.01
		Carbohydrate supplementation	9	−0.64	−1.16, − 0.11	56..8	
		Non-intervention	5	−0.30	−0.81, 0.21	70.6	
HOMA-IR	Participants’ age	Adult	9	−0.25	− 0.38, − 0.11	64.9	0.02
		Adult+Elderly	4	−0.07	−0.13, − 0.01	64.8	
	Participants’ health condition	Healthy	8	−0.07	− 0.13, − 0.01	59.6	< 0.001
		Chronic disease	5	−0.48	−0.70, − 0.26	42.2	
	Intervention type	Isolated	4	−0.20	−0.33, − 0.06	72.8	0.04
		Whey proteins	9	−0.07	− 0.13, − 0.01	64.1	
	Study duration	< 12 week	7	−0.04	−0.14, 0.06	80.2	< 0.18
		≥12 week	6	−0.12	−0.19, − 0.06	00.0	
	Sample size	n < 50	6	−0.11	−0.19, − 0.04	74.2	0.47
		n ≥ 50	7	−0.07	−0.16, 0.01	64.1	
	Type of control	Placebo product	2	−0.86	−1.43, − 0.29	00.0	< 0.01
		Carbohydrate supplementation	8	−0.22	−0.35, − 0.10	61.6	
		Non-intervention	3	−0.06	−0.12, 0.01	66.1	
TC	Participants’ age	Adult	12	−12.86	−16.11, −9.61	81.7	0.06
		Adult+Elderly	10	−9.07	−11.39, −6.74	96.1	
	Participants’ health condition	Healthy	15	−8.75	−10.87, −6.63	94.3	0.001
		Chronic disease	7	−16.40	−20.53, − 12.27	84.7	
	Intervention type	Isolated	7	−9.67	−12.52, −6.82	95.6	0.10
		Whey proteins	15	− 11.77	−14.43, −9.11	99.5	
	Study duration	< 12 week	10	−15.83	−18.33, − 13.34	96.1	< 0.001
		≥12 week	12	−3.01	−5.90, −0.12	40.6	
	Sample size	n < 50	14	−11.24	− 13.32, −9.15	95.3	0.04
		n ≥ 50	8	−6.21	−10.71, − 1.71	44.1	
	Type of control	Placebo product	2	−1.99	−10.44, 6.47	00.0	0.07
		Carbohydrate supplementation	16	−11.49	−13.76, −9.22	91.0	
		Non-intervention	4	−8.92	−12.63, −5.20	97.5	
TG	Participants’ age	Adult	11	−6.78	−10.71, −2.85	76.4	< 0.001
		Adult+Elderly	11	− 21.43	− 24.28, −18.58	94.2	
	Participants’ health condition	Healthy	15	−15.58	− 17.99, − 13.16	94.1	0.02
		Chronic disease	7	−25.04	−32.96, −17.11	1.0	
	Intervention type	Isolated	7	−13.90	− 16.94, 10.87	97.0	0.04
		Whey proteins	15	−19.86	−23.47, − 16.25	69.8	
	Study duration	< 12 week	9	−15.63	−18.60, − 12.66	96.2	0.43
		≥12 week	13	−17.52	−21.19, − 13.85	66.3	
	Sample size	n < 50	13	−17.05	−19.50, − 14.59	94.9	0.11
		n ≥ 50	9	−11.33	−18.06, −4.59	36.9	
	Type of control	Placebo product	2	−25.36	−41.44, −0.29	00.0	0.20
		Carbohydrate supplementation	16	−15.14	−17.90, − 12.38	93.9	
		Non-intervention	4	−18.81	−23.18, − 14.44	00.0	
LDL	Participants’ age	Adult	9	−1.73	−5.32, 1.87	47.1	0.001
		Adult+ Elderly	10	−8.9	−10.98, −6.81	96.9	
	Participants’ health condition	Healthy	14	− 8.31	− 10.25, − 6.36	95.7	0.001
		Chronic disease	5	0.45	−4.39, 5.29	20.7	
	Intervention type	Isolated	5	−10.49	−13.75, −7.22	88.7	< 0.01
		Whey proteins	14	−6.41	−8.67, −4.15	96.1	
	Study duration	< 12 week	8	−18.51	−21.39, − 15.62	96.1	< 0.001
		≥12 week	11	0.22	−2.09, 2.52	73.7	
	Sample size	n < 50	11	−5.88	−7.98, −3.79	94.9	0.02
		n ≥ 50	8	−10.56	−14.11, − 7.02	94.1	
	Type of control	Placebo product	2	4.36	−2.84, 11.56	54.4	< 0.01
		Carbohydrate supplementation	14	−7.75	−10.28, 5.23	81.3	
		Non-intervention	3	−7.98	−10.74, −5.22	99.2	
HDL	Participants’ age	Adult	11	−1.65	−2.41, −0.89	34.4	< 0.001
		Adult+ Elderly	10	1.40	1.10, 1.71	94.3	
	Participants’ health condition	Healthy	14	1.48	1.17, 1.79	94.5	< 0.001
		Chronic disease	7	−1.94	−2.68, −1.20	87.1	
	Intervention type	Isolated	6	1.57	1.25, 1.89	97.7	< 0.001
		Whey proteins	15	−1.15	−1.79, −0.51	83.6	
	Study duration	< 12 week	9	1.05	0.76, 1.35	97.6	0.13
		≥12 week	12	0.32	−0.58, 1.22	34.9	
	Sample size	n < 50	13	1.15	0.85, 1.44	96.3	< 0.001
		n ≥ 50	8	−0.66	−1.59, 0.28	56.0	
	Type of control	Placebo product	2	1.61	−0.09, 3.32	78.0	0.15
		Carbohydrate supplementation	16	1.04	0.74, 1.33	95.5	
		Non-intervention	3	0.03	−1.03, 1.09	70.6	

*HOMA IR* homeostasis model assessment of insulin resistance, *HbA1c* glycated hemoglobin, *TG* triglyceride, *TC* total cholesterol, *HDL-C* HDL-cholesterol, *LDL-C* LDL-cholesterol

### The effects of whey protein on serum lipoproteins

A significant reduction of triglycerides levels (18 studies with 22 effect sizes) (WMD: -17.12; 95% CI: − 26.52, − 7.72) (Table 2 & Fig. 2e) total cholesterol (18 studies with 22 effect sizes) (WMD: -10.88; 95% CI -18.60, − 3.17) (Table 2 & Fig. 2f), LDL-cholesterol (15 studies with 19 effect sizes) (WMD: -8.47% CI: − 16.59, − 0.36) (Table 2 & Fig. 2j) and total cholesterol/HDL-cholesterol (3 studies) (WMD: -0.26; 95% CI: − 0.41, − 0.10) (Table 2 & Fig. 2k) was found following the consumption of whey protein. Whey protein did not have any significant impact on HDL-cholesterol (17 studies with 21 effect sizes) (WMD: -0.13; 95% CI: − 1.74, 1.48) (Table 2 & Fig. 2h). The quality of evidence was low for triglycerides, total and LDL-cholesterol in GRADE system. While HDL-cholesterol had a very low evidence quality of evidence. For total cholesterol/HDL-cholesterol the quality of evidence was high (Supplemental file- Table 3). After adjustment, PI showed that results remained significant for triglycerides (95%PI: − 27.41, − 7.70), total cholesterol (95%PI: − 20.32, − 5.09), LDL-cholesterol (95%PI: − 15.96, − 0.51), and total cholesterol/HDL-cholesterol (95%PI: − 0.69, − 0.07), but this finding were insignificant for HDL-cholesterol (95%PI: − 1.90, 1.00).

Whey protein reduced triglycerides concentrations in all subgroups. In a subgroup analysis of total cholesterol, a significant change was seen in all subgroups except in studies which used placebo (WMD: -1.99; 95% CI: − 10.44, 6.47) **(**Table 3**)**. Whey protein intake did not have an effect on LDL-cholesterol levels in studies which were performed on adults (WMD: -1.73; 95% CI: − 5.32, 1.87), in studies done on patients with chronic diseases (WMD: 0.45; 95% CI -4.39, 5.29), and in studies with duration ≥12 weeks (WMD: 0.22; 95% CI: − 2.09, 2.52) or those which used placebo (WMD: 4.36; 95% CI: − 2.84, 11.56). Whey protein did not have an effect on HDL-cholesterol levels in some subgroups, including studies with duration ≥12 weeks (WMD: 0.32; 95% CI: − 0.58, 1.22) and sample size ≥50 (WMD: -0.66; 95% CI: − 1.59, 0.28) and in studies which used placebo (WMD: 1.61; 95% CI: − 0.09, 3.32) or non-intervention controls (WMD: 0.03; 95% CI: − 1.03, 1.09).

### Publication bias and sensitivity analysis

Publication bias was investigated for outcomes with at least 10 related studies, including FBS, TC, TG, LDL, and HDL. Visual inspection of funnel plots showed no significant publication bias for the included studies (Supplemental file- Fig. 1A-J). This finding was also confirmed by the Eggers’ regression test (For FBS: *P* = 0.05; for TC: *P* = 0.74; for TG: *P* = 0.81; for LDL: 0.44; for HDL: 0.37). Sensitivity analysis also showed that no specific study had great influence on the overall findings of the study (Supplemental file- Fig. 2A-E).

### Meta-regression

Dose-response analysis for the influence of study duration on the association between whey protein supplementation and outcomes of interest was measured using meta-regression. This analysis did not show any significant dose-response association between study duration and changes in FPG (*P* = 0.79), HOMA-IR (*P* = 0.36), HbA1C (*P* = 0.49), total cholesterol (*P* = 0.43), triglycerides (*P* = 0.22), LDL-cholesterol (*P* = 0.27), and HDL-cholesterol (*P* = 0.62) concentrations. However, a marginally significant inverse association was found between study duration and changes in insulin concentrations (P = 0.05). This means that reduction in insulin concentration following whey protein supplementation was more considerable in studies with longer intervention period.

## Discussion

For the first time, this meta-analysis analyzed whey protein effects on serum lipoproteins and parameters of glucose homeostasis in patients with MetS and related disorders. It indicated that whey protein might improve insulin, HOMA-IR, HbA1c triglycerides, total cholesterol, LDL-cholesterol and total cholesterol/HDL-cholesterol ratio in MetS and related disorders, but it had no effects on HDL-cholesterol and FPG levels.

### Whey protein and glucose metabolism

This meta-analysis suggested that whey protein significantly decreased the levels of insulin as well as HOMA-IR and HbA1c, but did not have any effect on FPG levels. In the present study, subgroup analyses based on sample size, duration and health condition showed a significant reduction in FPG levels. However, after PI estimation, results were insignificant for all parameters of glycemic control which maybe reflective of the variation in settings and treatment effects. Previously, some epidemiological studies have demonstrated that consumption of milk and/or dairy products was correlated with a lower risk of metabolic changes and CVD. In particular, whey protein intake seems to improve metabolic parameters due to bioactive substances, including immunoglobulins, glutamine, lactoferrin and lactalbumin. It is also an excellent source of BCAAs. However, results of different studies are conflicting. Whey protein supplementation has been suggested for both prevention and treatment of obesity and diabetes in humans and in animal models [42]. One of the reasons could be the reduction of the long and short term appetite [43]. In a study by Rigamonti et al. [44], taking whey proteins improved glucometabolic homeostasis in young obese women. Two recent meta-analyses including studies on overweight and obese participants, have indicated that whey protein administration might improve FPG levels [11, 45]. Taking whey proteins during 12 weeks in overweight and obese individuals significantly improved their insulin levels and decreased total cholesterol and LDL-cholesterol levels [10]. However, the consumption of 125 mL/day of a milk drink supplemented with whey peptides for 12 weeks by mildly hypertensive subjects did not improve metabolic parameters such as FPG, insulin and serum lipids [22]. In subjects with PCOs, a hypocaloric diet plus whey protein did not affect glycemic control [24]. Low fat high-casein or whey protein rich weight maintenance diets had not adverse effects on metabolic parameters and markers of cardiovascular risk in moderately obese patients without metabolic or cardiovascular complications while reduced their weight [26]. Whey protein may be involved in decreasing postprandial hyperglycemia and could improve the insulin response by different mechanisms. After its digestion, a rapid increase in amino acids (BCAAs, in particular) results in increased insulin release which probably improves postprandial hyperglycemia. Bioactive peptides also activate the release of incretin hormones including GIP and GLP-1 which have an important role in improvement of insulin resistance. On the other hand, peptides from hydrolyzation of whey inhibit dipeptidyl peptidase-IV and inhibit degradation of GIP and GLP-1 [46]. Based on all these results as well as this study, short-term insulinotrophic effect of whey proteins may be a beneficial in the management of MetS and/or T2DM.

### Whey protein and serum lipoproteins

This meta-analysis showed that whey protein decreased triglycerides, total cholesterol, LDL-cholesterol and total cholesterol/HDL-cholesterol ratio in patients with MetS and its components, but did not have any effect on HDL-cholesterol levels. In the present study, the reduction of triglycerides was significant in all subgroups and total cholesterol also significantly reduced in the most subgroups. HDL-cholesterol levels also were increased in some subgroup analyses such as studies used carbohydrate supplementation as control which may represent that the using of certain control may affect the findings of studies regarding the efficacy of whey protein supplement on HDL-cholesretol levels. Increase in HDL-cholesterol levels was significant in studies conducted among adult and elderly populations, individuals without chronic diseases and studies with less than 12 weeks’ duration or with less than 50 participants. PI estimation, did not affect the significance of results for lipid profiles. Recently, a meta-analysis by Badely et al. [45] has been done to explore the effects of whey protein supplementation in overweight and obese subjects. The results indicated that whey protein supplementation when compared with different kind of controls caused a significant reduction in triglycerides and HDL-cholesterol in this population. However, a significant heterogeneity has been reported for these parameters. In another meta-analysis by Zhang et al. [47], whey protein intake also significantly decreased triglycerides levels and had no effects on total cholesterol, LDL- and HDL-cholesterol but the subgroup analyses showed that significant reduction of triglycerides disappeared in several cases including lower dosage of whey protein, low BMI groups of participants, exercise performing and energy restriction during the trial. In a study by Fekete et al. [31], the consumption of unhydrolyzed milk proteins (56 g/day) during 8 weeks in subjects with prehypertension and mild HTN decreased serum triglycerides, and improved biomarkers of endothelial function and vascular reactivity. Moderate-high doses of whey protein during 16 weeks significantly reduced total cholesterol/HDL-cholesterol ratio in obese men [37]. Fortified yogurt with whey protein during 10-week significantly reduced triglycerides levels in patients with MetS [9]. As mentioned before, supplementation with whey proteins during 12 weeks in overweight and obese subjects was associated with a significant decrease in total cholesterol and LDL-cholesterol [10]. In another study, a 12-week supplementation with whey protein in subjects with prehypertension and mildly hypertensive patients did not have any significant effect on serum lipoproteins [40]. Calcium intake from dairy products has been correlated with calcium-fatty acid soap production in the gut, which in turn results in decreased fat absorption [48] Therefore, calcium intake from whey protein may be responsible for the lipid-lowering effects of this protein. Different proteins from different sources and qualities could cause different metabolic effects [49, 50]. Whey protein intake might have effects on lipid metabolism by inhibition of cholesterol absorption in the intestine mediated by its functional components like beta-lactoglobulin and sphingolipids. In addition, other lipid lowering mechanisms like stimulation of lipoprotein lipase, and down-regulation of gene expression important for cholesterol absorption and fatty acid transport have been associated to BCAAs content of whey protein [51–54].

### Study strengths and limitations

This study is a comprehensive systematic review and meta-analysis of studies about the effect of whey protein supplementation on serum levels of several metabolic parameters. Previous meta-analyses focused on the metabolic effects of whey protein in obese and overweight individuals, while this meta-analysis has been done on studies in patients with MetS and related disorders. However, this study has some limitations. First, whey protein was used in different dosages in the included studies. Moreover, study duration and control group were varied between included studies. We tried to minimize these discrepancies by different subgroup analyses. Intervention period was limited in all the included studies. Therefore, RCTs with longer duration are needed to determine clearly the effects of whey protein supplementation on metabolic parameters in moderate to long-term interventions. The limited sample size of included studies was another limitation. In addition, most included studies were done in Western countries and only limited data are available from Asian and Australian populations. In addition, included studies suffer from different sources of bias in some aspects and this should be taken into consideration. Also, due to various regimens, doses, duration, center settings, populations and sample size the results of present study should be interpreted with cautious. Therefore, further large-scale studies on different populations are required to provide some clear answers concerning.

## Conclusions

This meta-analysis indicated potential effects of whey protein on improving HbA1c, insulin, HOMA-IR, triglycerides, total cholesterol, LDL-cholesterol and total/HDL-cholesterol ratio in patients with MetS and related disorders, but it did not show any effect on HDL-cholesterol, and FPG levels. In the present study, the significance of findings for parameters of glycemic status were disappeared after PI estimation, which may be due to the heterogeneity. Therefore, the efficacy of whey protein supplementation on glycemic control should be identified in future studies. In order to overcome different sources of bias future RCTs need to be designed with appropriate blinding, allocation concealment and data report to overcome different sources of bias.

## Supplementary information

**Additional file 1: Table 1**. Search strategies and the number of publications in each electronic database. **Table 2**. Cochrane quality assessment of the included studies. **Table 3**. GRADE summary of findings.

**Additional file 2: Fig. 1**A-J. Funnel plots for A) FPG, B) insulin, C) HOMA-IR, D) triglycerides, E) total cholesterol, F) LDL-cholesterol and J) HDL-cholesterol. **Fig. 2**A-E. Funnel plots for A) FPG, B) triglycerides, C) total cholesterol, D) LDL-cholesterol and E) HDL-cholesterol.

## Data Availability

The primary data for this study is available from the authors on direct request.

## Abbreviations

HOMA IR: Homeostasis model assessment of insulin resistance
HbA1c: Glycated hemoglobin
TG: Triglycerides
TC: Total cholesterol
HDL-C: HDL-cholesterol
LDL-C: LDL-cholesterol
