# Supplementary material for: Effects of whey protein on glycemic control and serum lipoproteins in patients with metabolic syndrome and related conditions: a systematic review and meta-analysis of randomized controlled clinical trials
Source: Lipids Health Dis. 2020 Sep 21;19:209. doi: 10.1186/s12944-020-01384-7 (PMC7504833; doi:10.1186/s12944-020-01384-7)
Supplement: Supplementary file 1 — Additional file 1: Table 1. Search strategies and the number of publications in each electronic database. Table 2. Cochrane quality assessment of the included studies. Table 3. GRADE summary of findings. [file 12944_2020_1384_MOESM1_ESM.docx]

**Supplemental file- Table 1.** Search strategies and the number of publications in each electronic database.

| Database | Search strategy | Number of records |
| --- | --- | --- |
| PubMed | ("whey protein"[MeSH] OR "whey protein*"[tiab] OR whey[tiab]) AND ("glycemic control"[Tiab] OR glucose[MeSH] OR glucose[tiab] OR "fasting plasma glucose"[Tiab] OR FPG[Tiab] OR FBG[Tiab] OR FBS[Tiab] OR insulin[MeSH] OR insulin[Tiab] OR "Glycated Hemoglobin A"[MeSH] OR "Glycated Hemoglobin"[tiab] OR HbA1c[Tiab] OR "homeostatic model of insulin resistance"[Tiab] OR “HOMA-IR”[Tiab] OR "lipid profile*"[Tiab] OR triglycerides[MeSH] OR triglyceride*[Tiab] OR triacylglycerol[tiab] OR cholesterol[MeSH] OR cholesterol[Tiab] OR VLDL[Tiab] OR LDL[Tiab] OR HDL[Tiab]) AND (“metabolic syndrome”[Mesh] OR “metabolic syndrome*”[tiab] OR “metabolic disorders*”[tiab] OR overweight[MeSH] OR overweight[tiab] OR obesity[Mesh] OR obesity[MeSH] OR obese[tiab] OR diabetes[MeSH] OR diabetes[tiab] OR diabetic[tiab] OR “cardiovascular disease*”[MeSH] OR “cardiovascular disease*”[tiab] OR cardiovascular[tiab] OR “cardiovascular disorder*”[tiab] OR CVD[tiab] OR atherosclerosis[tiab] OR coronary[tiab] OR dyslipidemia[MeSH] OR hyperlipidemia[MeSH] OR “blood pressure”[MeSH] dyslipidemia[tiab] OR hyperlipidemia[tiab] OR “blood pressure”[tiab] OR hypertension[MeSH] OR hypertension[tiab] OR HTN[tiab] OR "coronary artery disease*”[MeSH] OR "coronary artery disease*”[tiab] OR CAD[tiab] OR “non-alcoholic fatty liver disease”[Mesh] OR “non-alcoholic fatty liver disease”[tiab] OR NAFLD[tiab]). | 61 |
| Scopus | TITLE-ABS-KEY("whey protein" OR whey) AND TITLE-ABS-KEY("glycemic control" OR glucose OR "fasting plasma glucose" OR FPG OR FBG OR FBS OR insulin OR "Glycated Hemoglobin A" OR “HbA1c” OR "homeostatic model of insulin resistance" OR “HOMA-IR” OR "lipid profile*" OR triglycerides OR triacylglycerol OR cholesterol OR VLDL OR LDL OR HDL) AND TITLE-ABS-KEY(“metabolic syndrome*” OR “metabolic disorders*” OR overweight OR obesity OR obese OR diabetes OR diabetic OR “cardiovascular disease*” OR CVD OR dyslipidemia OR hyperlipidemia OR “blood pressure” OR hypertension OR HTN OR "coronary artery disease*” OR “CAD” OR “non-alcoholic fatty liver disease” OR “NAFLD” OR atherosclerosis OR coronary). | 449 |
| Web of Science | ALL=("whey protein" OR whey) AND ALL=("glycemic control" OR glucose OR "fasting plasma glucose" OR FPG OR FBG OR FBS OR insulin OR "Glycated Hemoglobin A" OR “HbA1c” OR "homeostatic model of insulin resistance" OR “HOMA-IR” OR "lipid profile*" OR triglycerides OR triacylglycerol OR cholesterol OR VLDL OR LDL OR HDL) AND ALL=(“metabolic syndrome*” OR “metabolic disorders*” OR overweight OR obesity OR obese OR diabetes OR diabetic OR “cardiovascular disease*” OR CVD OR dyslipidemia OR hyperlipidemia OR “blood pressure” OR hypertension OR HTN OR "coronary artery disease*” OR “CAD” OR “non-alcoholic fatty liver disease” OR “NAFLD” OR atherosclerosis OR coronary) | 544 |
| Cochrane | ("whey protein" OR whey) AND ("glycemic control" OR glucose OR "fasting plasma glucose" OR FPG OR FBG OR FBS OR insulin OR "Glycated Hemoglobin A" OR “HbA1c” OR "homeostatic model of insulin resistance" OR “HOMA-IR” OR "lipid profile*" OR triglycerides OR triacylglycerol OR cholesterol OR VLDL OR LDL OR HDL) AND (“metabolic syndrome*” OR “metabolic disorders*” OR overweight OR obesity OR obese OR diabetes OR diabetic OR “cardiovascular disease*” OR CVD OR dyslipidemia OR hyperlipidemia OR “blood pressure” OR hypertension OR HTN OR "coronary artery disease*” OR “CAD” OR “non-alcoholic fatty liver disease” OR “NAFLD” OR atherosclerosis OR coronary) | 262 |

| **Supplemental file- Table 2**. Cochrane quality assessment of the included studies. | | | | | | | |
| --- | --- | --- | --- | --- | --- | --- | --- |
| Study Ref. (year) | Random Sequence Generation | Allocation concealment | Blinding of participants and personnel | Blinding of outcome assessment | Incomplete outcome data | Selective reporting | Other sources of bias |
| Lee et al.[22] (2007) | U | L | L | U | L | L | L |
| Frestedt et al.[29] (2008) | U | L | U | U | L | U | H |
| Kasim-Karakas et al.[40] (2009) | L | L | L | H | L | L | L |
| Denysschen et al.[32] (2009) | U | U | L | U | L | L | U |
| Claessens et al.[34] (2009) | L | H | H | H | L | L | U |
| Pal et al.[10] (2010) | L | L | L | U | L | U | L |
| Sheikholeslami Vatani and Ahmadi Kani Golzar [37] (2012) | U | L | L | H | L | L | L |
| Petyaev et al.[23] (2012) | U | L | U | U | L | H | U |
| Tovar et al.[30] (2012) | L | U | U | U | L | L | L |
| Ormsbee et al.[33] (2015) | L | L | L | L | L | L | U |
| Fekete et al.[24] (2016) | L | L | L | L | L | L | L |
| Tovar et al.[31] (2016) | L | H | H | U | L | L | L |
| Jakubowicz et al.[28] (2017) | L | H | H | H | L | L | L |
| Lopes Gomes et al.[39] (2017) | L | H | H | H | L | L | L |
| Kjølbæk et al.[35] (2017) | L | L | L | U | L | L | L |
| Watson et al.[26] (2018) | L | L | L | H | L | H | L |
| Kemmler et al.[41] (2018) | L | U | H | L | L | H | L |
| Gaffney et al.[27] (2018) | L | L | L | U | L | H | L |
| Larsen et al.[38] (2018) | L | L | L | H | L | L | L |
| Mohammadi-Sartang et al.[9] (2018) | L | L | L | L | L | L | L |
| Yang et al.[25] (2019) | L | U | U | U | L | L | L |
| Rakvaag et al.[36] (2019) | L | L | L | L | L | L | L |
| U; unclear risk of bias, L; low risk of bias, H; high risk of bias. | | | | | | | |

| **Supplemental file- Table 3**. GRADE summary of findings. | | | |
| --- | --- | --- | --- |
| Outcome | Number of participants (trials) | Effect (95% CI) | Quality of evidence |
| FPG | 850 (17) | -0.61 (-2.83, 1.62) | Low  Due to impression and inconsistency |
| HbA1C | 691 (12) | -0.15 (-0.29, -0.01) | Moderate  Due to inconsistency |
| Insulin | 651 (12) | -0.94 (-1.68, -0.21) | Moderate  Due to inconsistency |
| HOMA-IR | 304 (6) | -0.20 (-0.36, -0.05) | Low  Due to inconsistency and high risk of bias |
| TG | 939 (18) | -17.12 (-26.52, -7.72) | Low  Due to inconsistency and high risk of bias |
| TC | 901 (18) | -10.88 (-18.60, -3.17) | Low  Due to inconsistency and high risk of bias |
| LDL | 830 (15) | -8.47 (-16.59, -0.36) | Low  Due to inconsistency and high risk of bias |
| HDL | 872 (17) | -0.13 (-1.74, 1.48) | Very low  Due to impression, inconsistency and high risk of bias |
| TC/HDL | 161 (3) | -0.26 (-0.41, -0.10) | High |
| HOMA IR, homeostasis model assessment of insulin resistance; HbA1c, glycated hemoglobin; TG, triglycerides; TC, total cholesterol, HDL-C, HDL-cholesterol; LDL-C, LDL-cholesterol. | | | |
