# Supplementary material for: Effects of whey protein on glycemic control and serum lipoproteins in patients with metabolic syndrome and related conditions: a systematic review and meta-analysis of randomized controlled clinical trials
Source: Lipids Health Dis. 2020 Sep 21;19:209. doi: 10.1186/s12944-020-01384-7 (PMC7504833; doi:10.1186/s12944-020-01384-7)
Supplement: Supplementary file 2 — Additional file 2: Fig. 1A-J. Funnel plots for A) FPG, B) insulin, C) HOMA-IR, D) triglycerides, E) total cholesterol, F) LDL-cholesterol and J) HDL-cholesterol. Fig. 2A-E. Funnel plots for A) FPG, B) triglycerides, C) total cholesterol, D) LDL-cholesterol and E) HDL-cholesterol. [file 12944_2020_1384_MOESM2_ESM.ppt]

## Slide 1
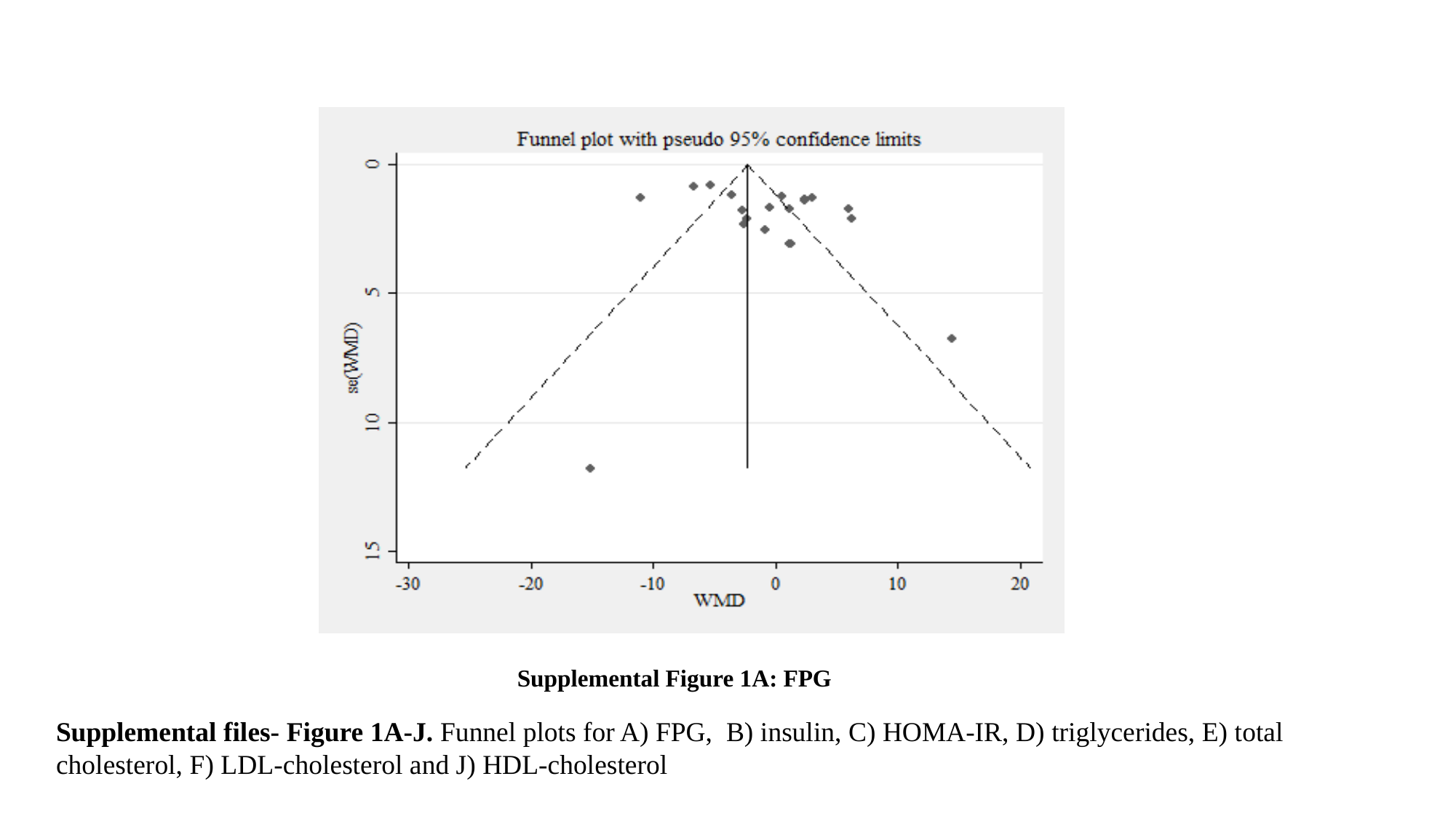

# Supplemental Figure 1A: FPG
Supplemental files- Figure 1A-J. Funnel plots for A) FPG, B) insulin, C) HOMA-IR, D) triglycerides, E) total cholesterol, F) LDL-cholesterol and J) HDL-cholesterol

## Slide 2
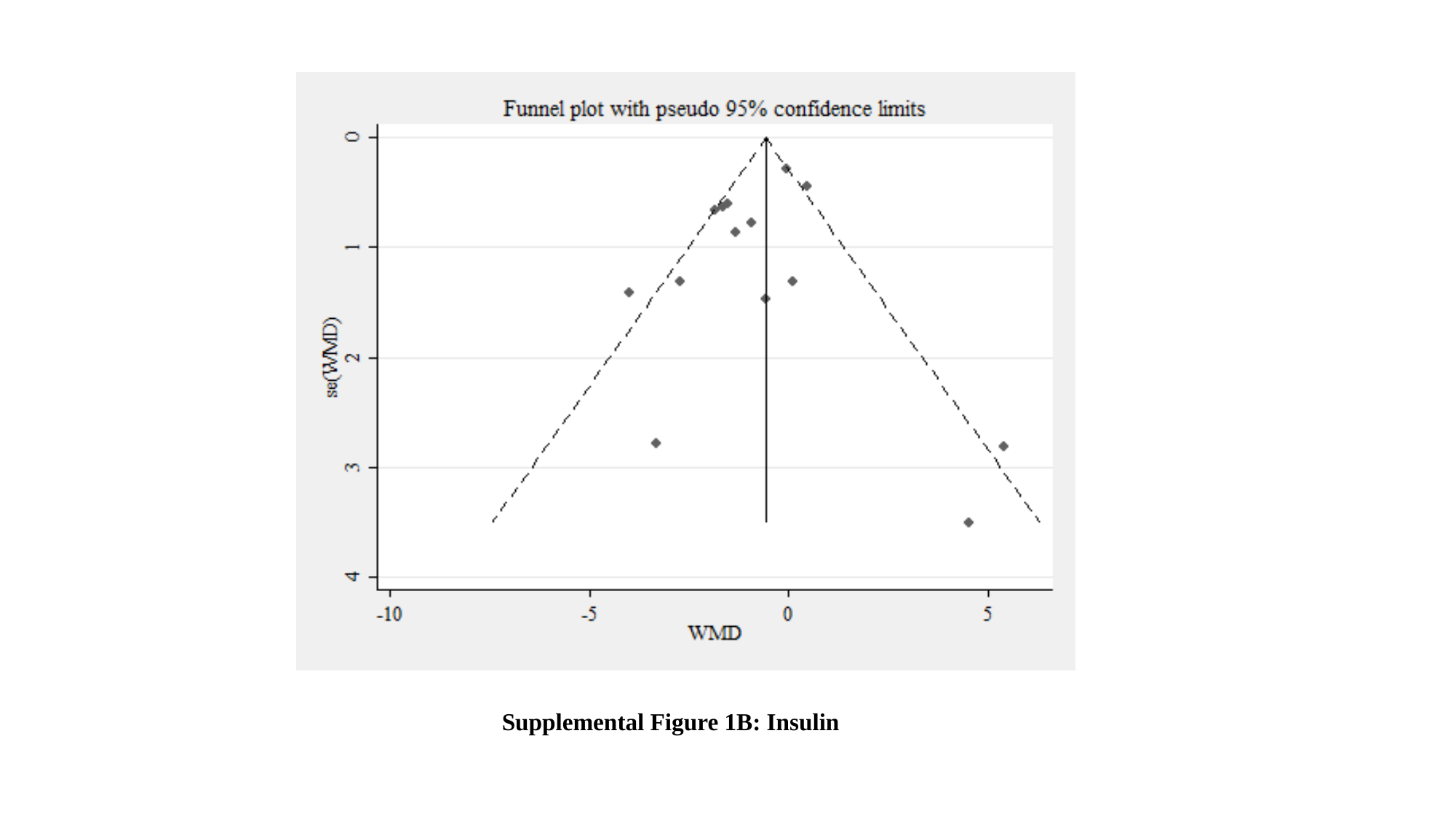

Supplemental Figure 1B: Insulin

## Slide 3
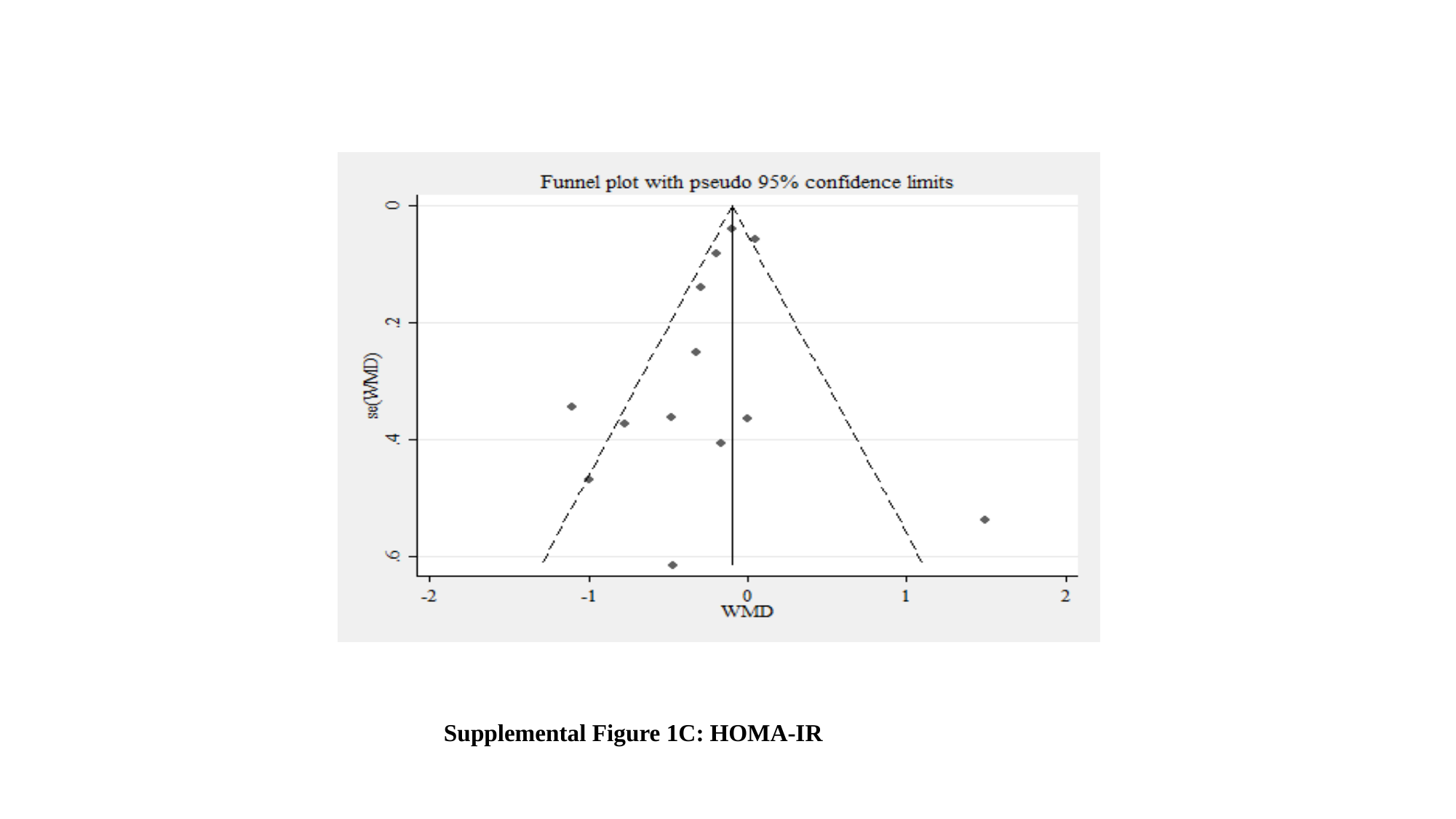

Supplemental Figure 1C: HOMA-IR

## Slide 4
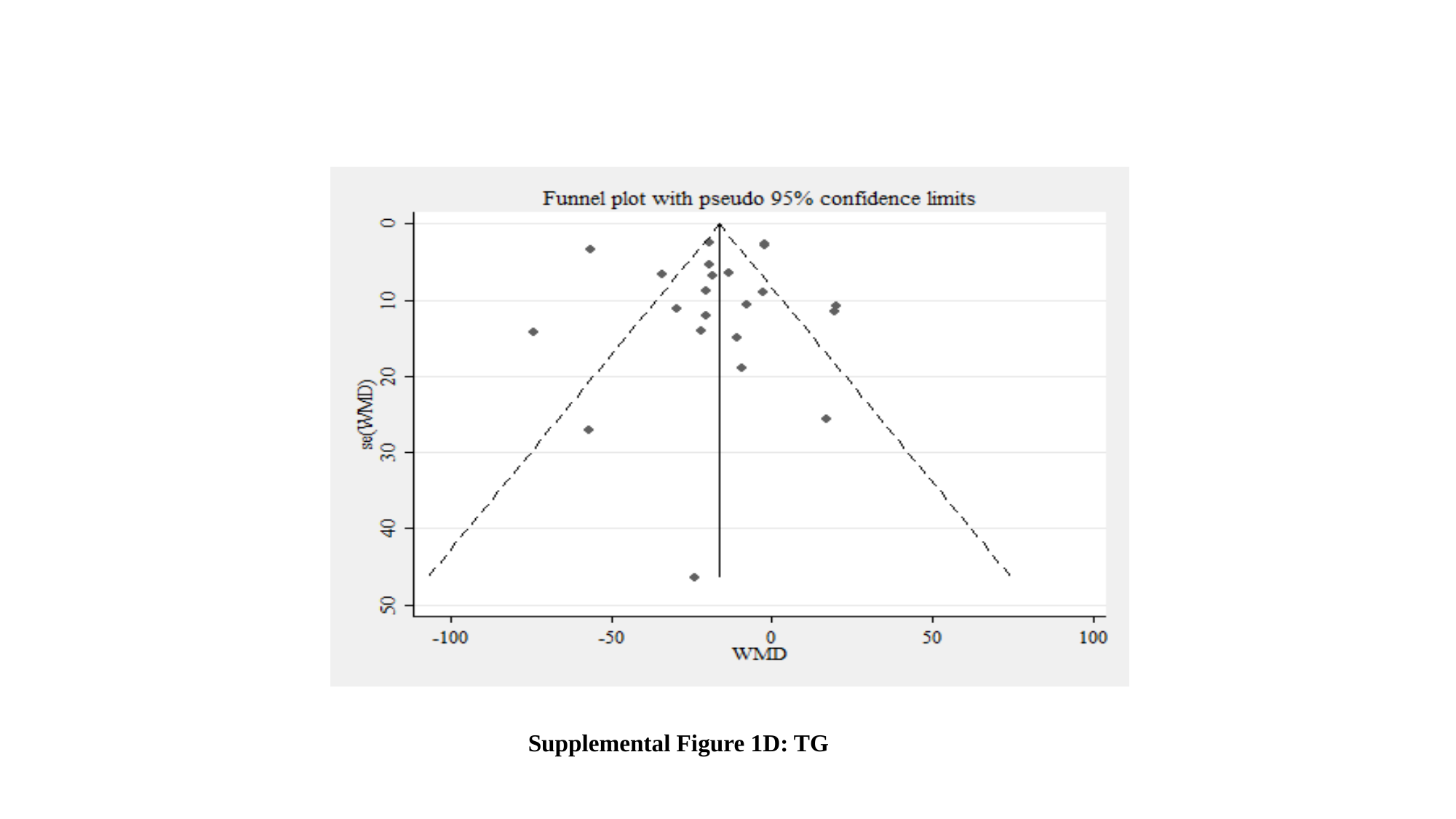

Supplemental Figure 1D: TG

## Slide 5
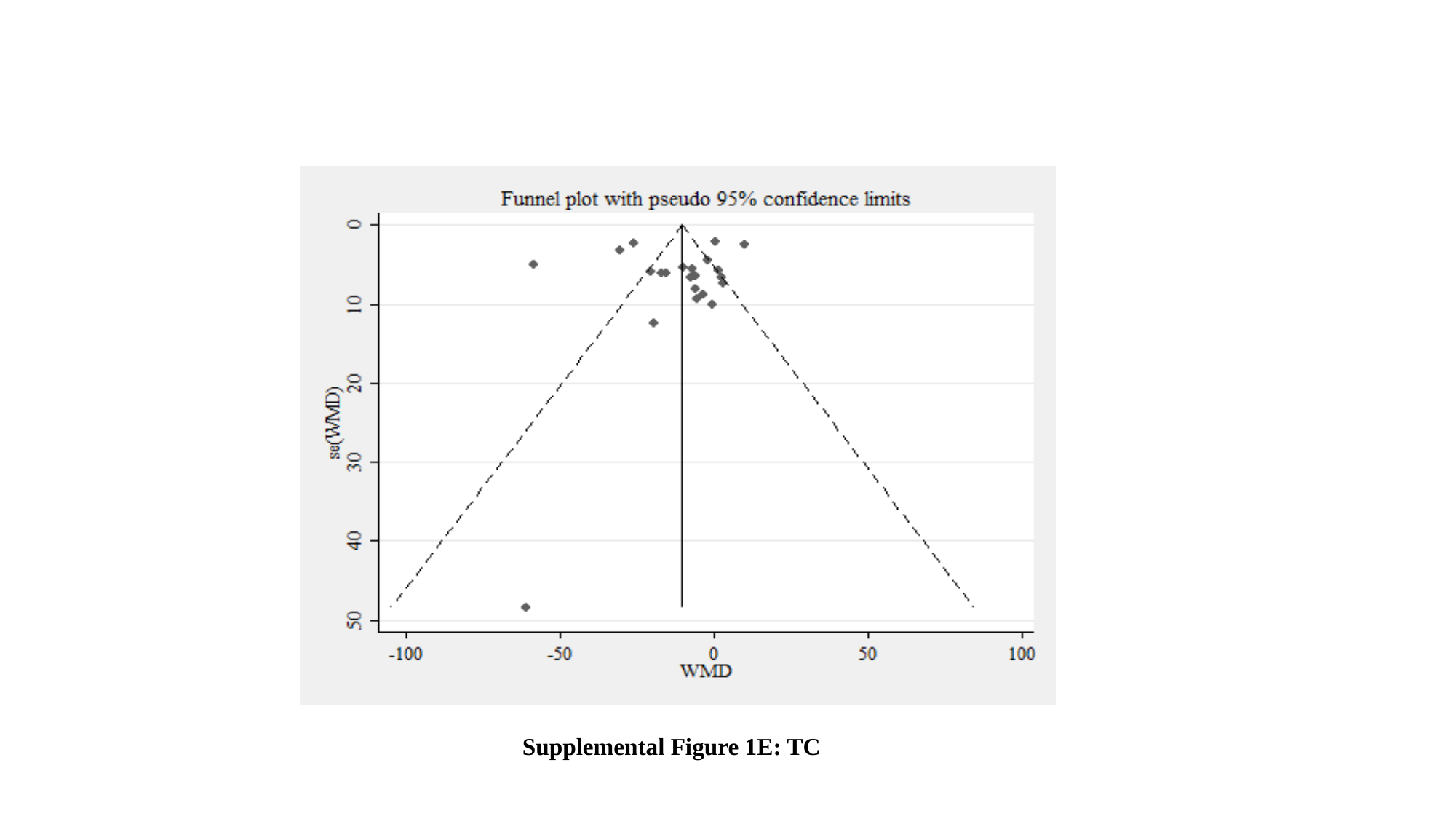

Supplemental Figure 1E: TC

## Slide 6
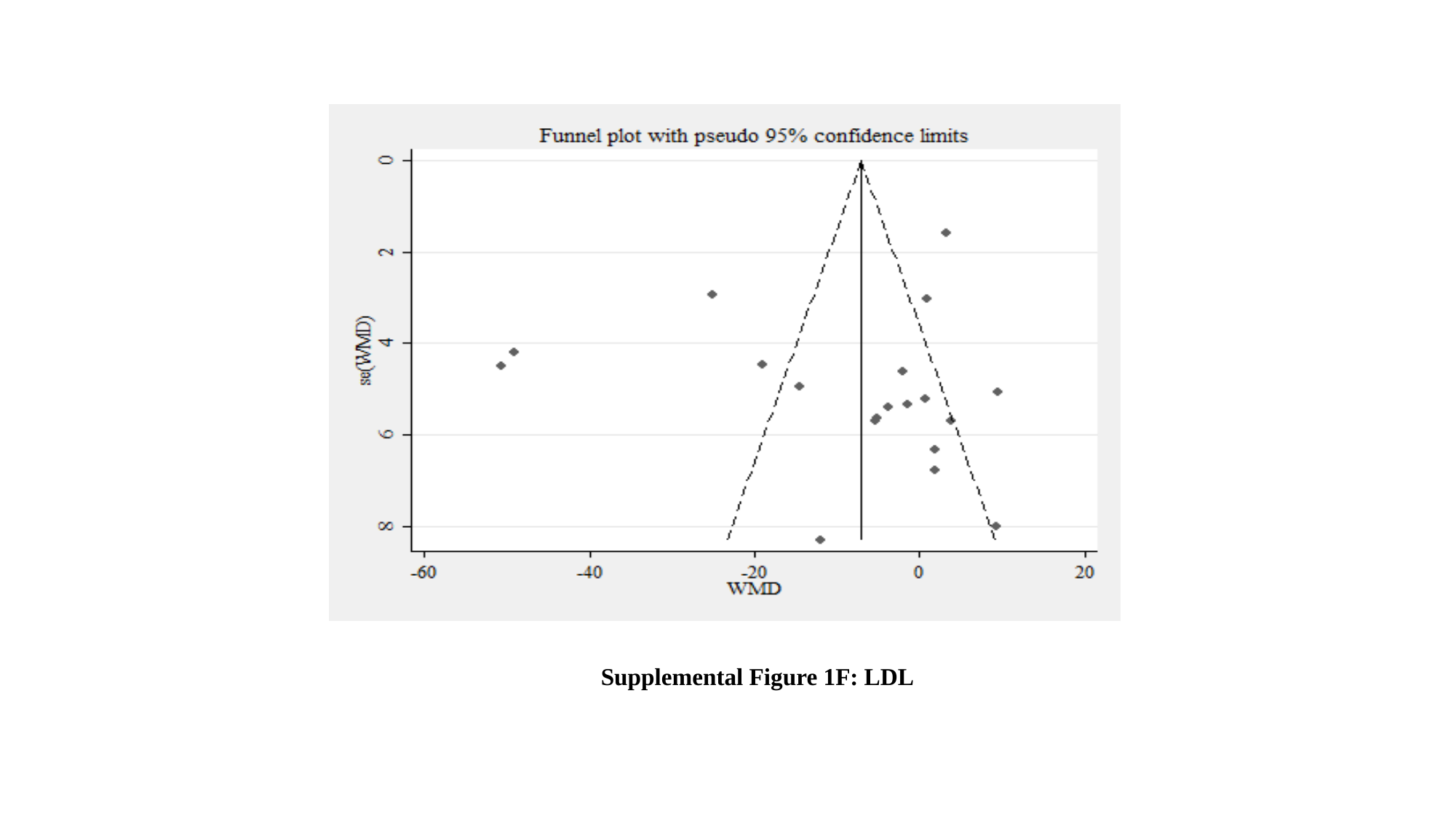

Supplemental Figure 1F: LDL

## Slide 7
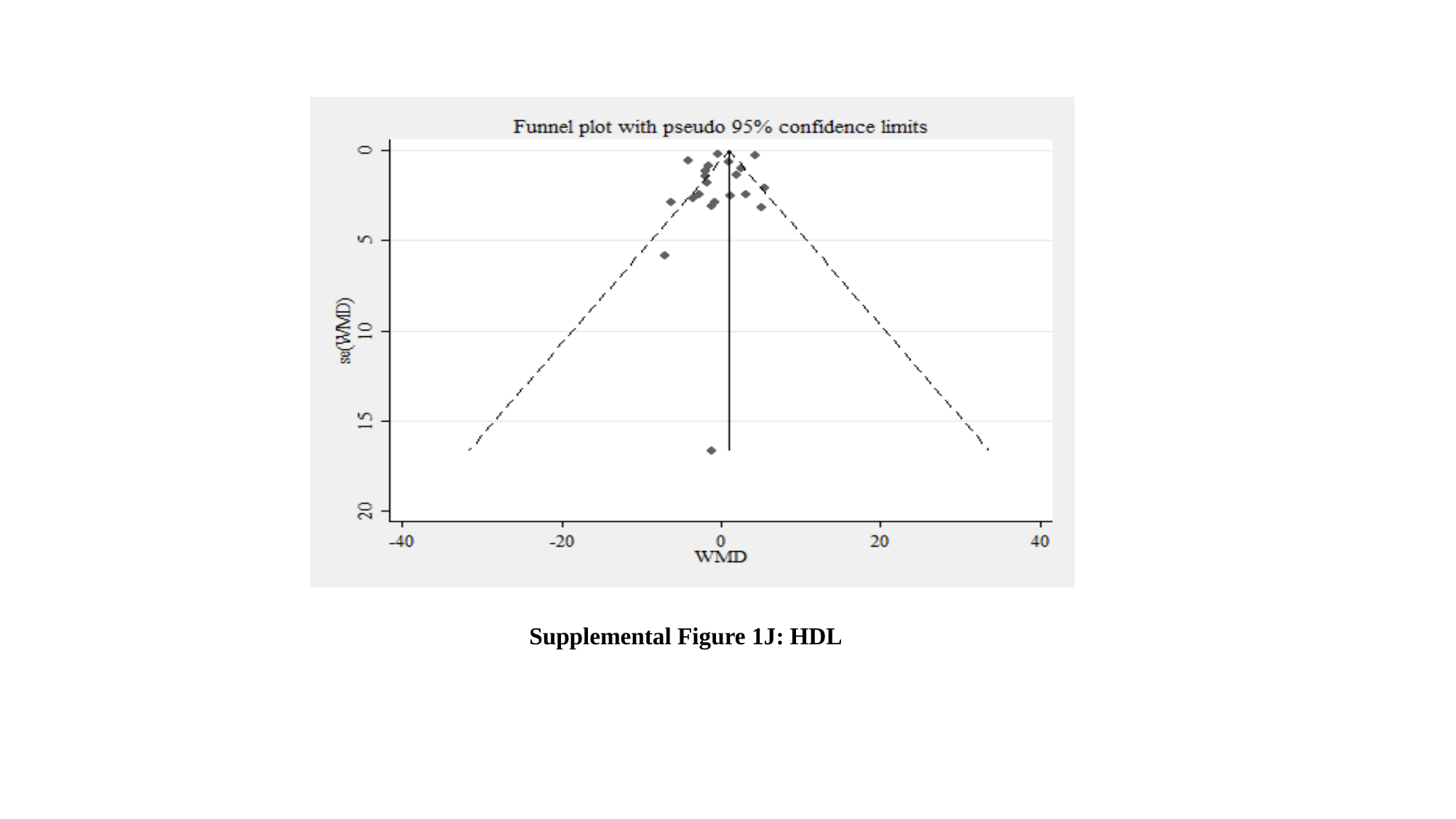

Supplemental Figure 1J: HDL

## Slide 8
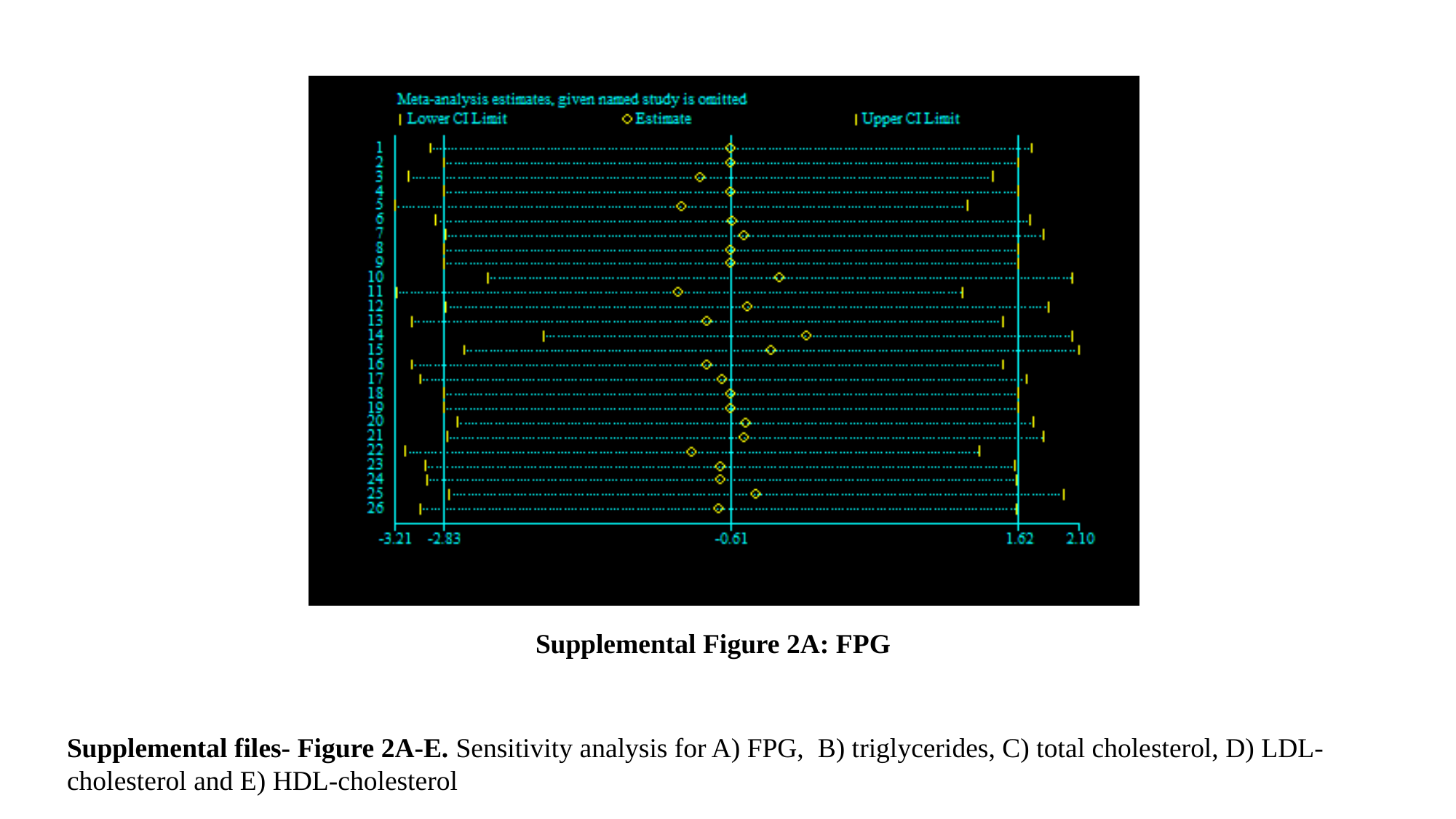

Supplemental Figure 2A: FPG
Supplemental files- Figure 2A-E. Sensitivity analysis for A) FPG, B) triglycerides, C) total cholesterol, D) LDL-cholesterol and E) HDL-cholesterol

## Slide 9
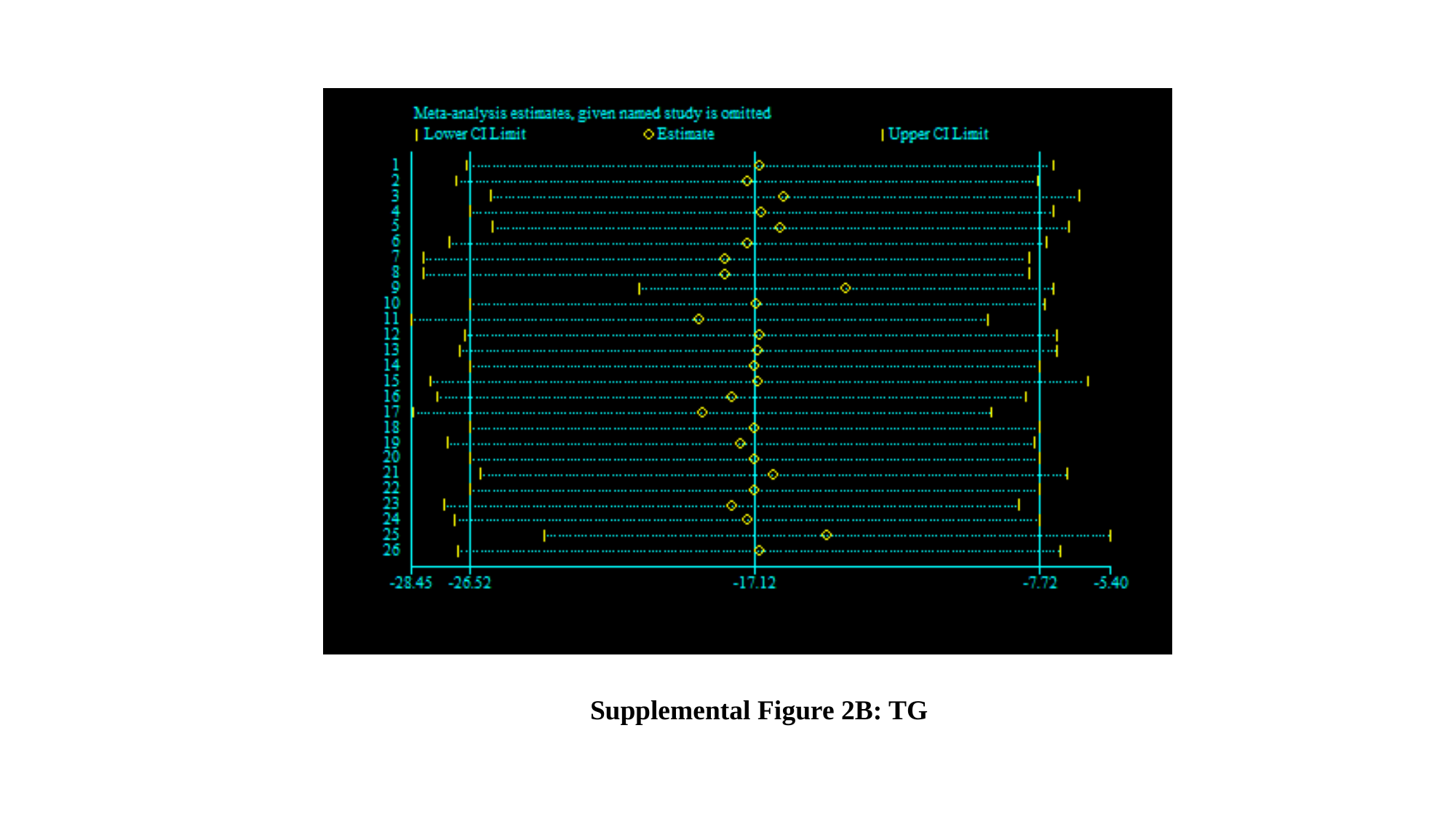

Supplemental Figure 2B: TG

## Slide 10
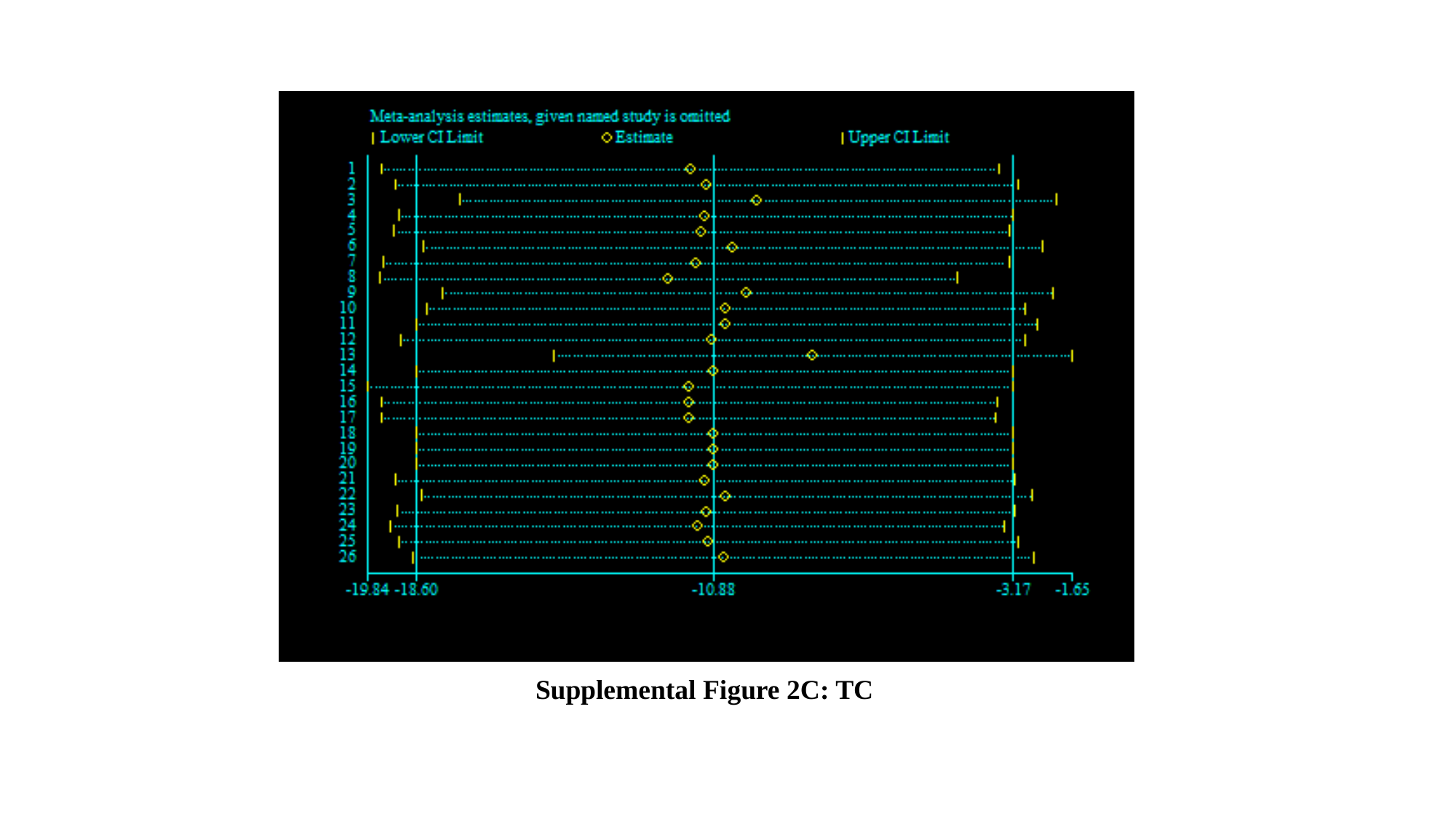

Supplemental Figure 2C: TC

## Slide 11
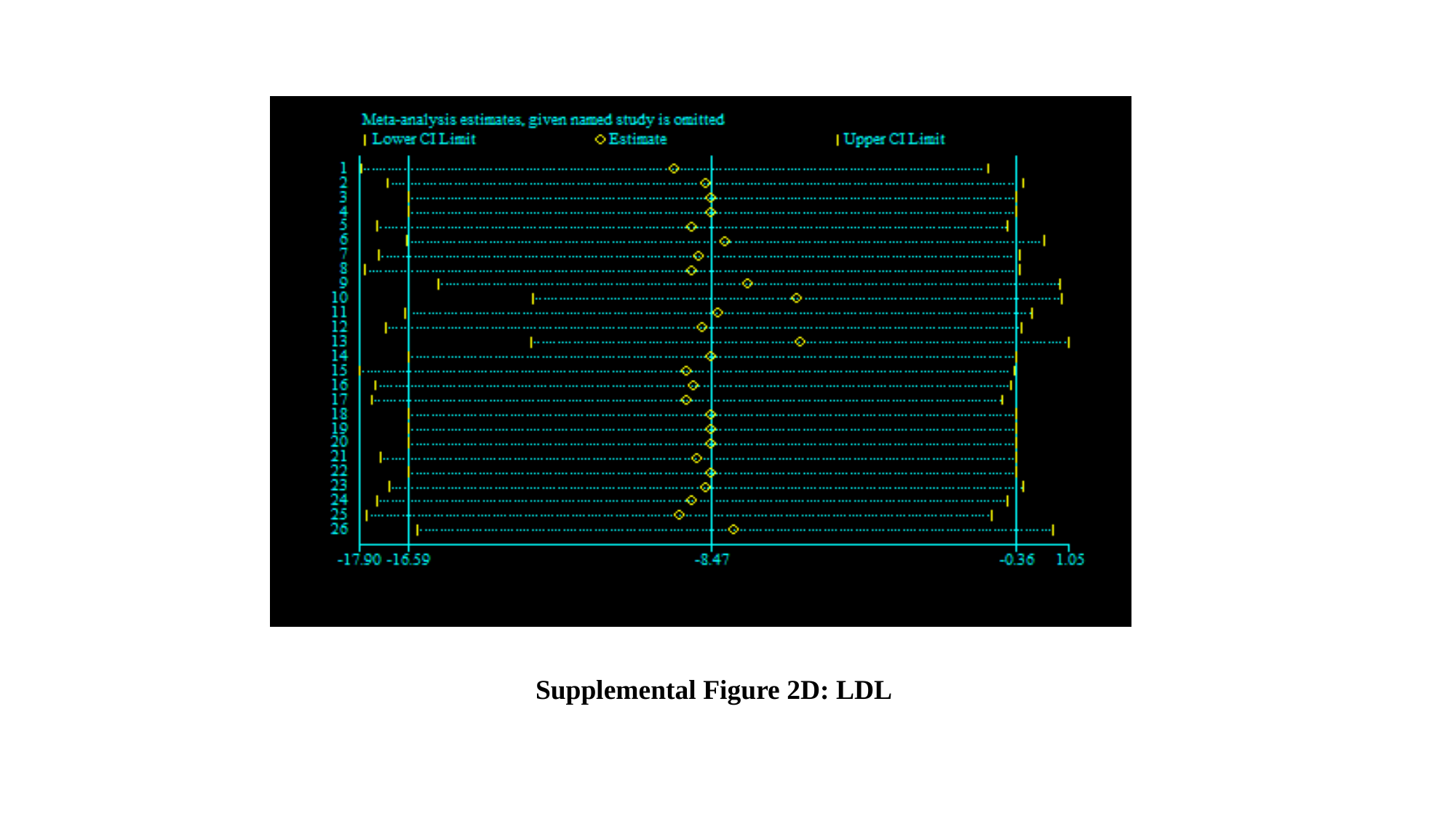

Supplemental Figure 2D: LDL

## Slide 12
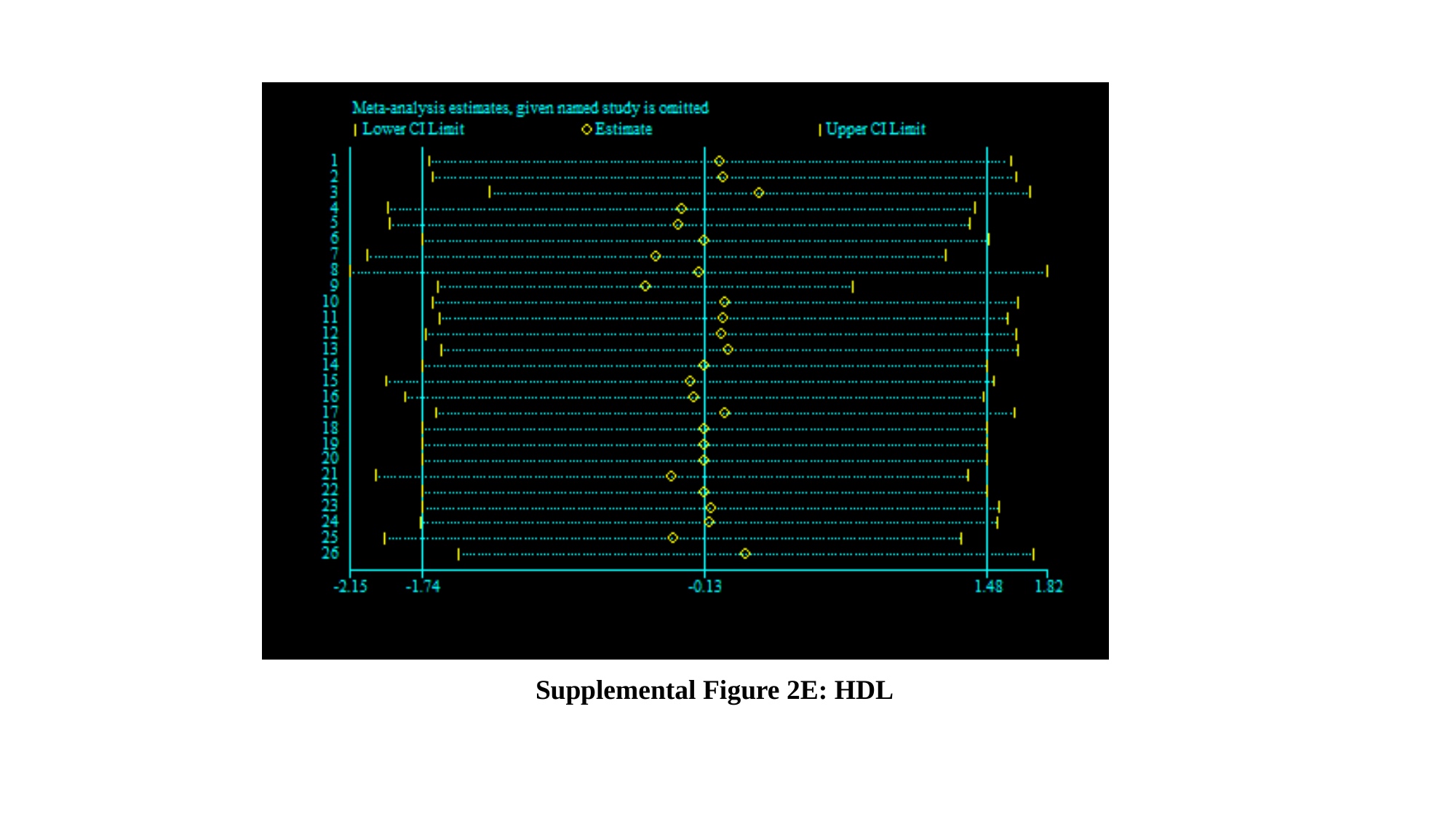

Supplemental Figure 2E: HDL
